# Hygrometrically controlled programmed cell death drives anther opening and pollen release

**DOI:** 10.1073/pnas.2420132122

**Published:** 2025-05-16

**Authors:** Anna Kampová, Moritz K. Nowack, Matyáš Fendrych, Stanislav Vosolsobě

**Affiliations:** ^a^Department of Experimental Plant Biology, Faculty of Science, Charles University, Prague 128 00, Czechia; ^b^Department of Plant Biotechnology and Bioinformatics, Ghent University, Ghent 9052, Belgium; ^c^VIB (Vlaams Instituut voor Biotechnologie), Center of Plant Systems Biology, Ghent 9052, Belgium

**Keywords:** anther dehiscence, pollen, humidity, stomata, programmed cell death

## Abstract

Anther dehiscence is the process that facilitates pollen release from mature anthers in flowering plants. Despite its crucial importance to reproduction, the underlying developmental mechanism and its integration with environmental cues remain poorly understood. Establishing noninvasive, controlled humidity treatments of *Arabidopsis thaliana* flowers, we show here that high humidity prevents anthers from opening. Manipulation of stomatal densities alters dehiscence dynamics, suggesting a contribution of controlled transpiration. Furthermore, analyses of subcellular markers revealed the occurrence of a developmentally prepared and environmentally triggered programmed cell death (PCD) process in specific anther tissues, epidermis and endothecium. Notably, genetic inhibition of PCD delays anther dehiscence, whereas precocious PCD induction promotes it. Our data reveal a rapid PCD execution process modulated by ambient humidity as instrumental for timely pollen release in the flowering plant *Arabidopsis*.

## Significance

Anther dehiscence, the process of opening the anthers to release pollen in flowering plants, is essential for reproductive success. Our study demonstrates that high humidity conditions delay anther opening by postponing programmed cell death (PCD) in specific anther tissues. By revealing how plants respond to environmental humidity to modulate anther dehiscence, our findings provide insights into the regulation of pollen release timing. This work contributes to understanding how plants synchronize developmental processes with external conditions, with potential implications for improving crop yield under varying climatic conditions.

Anther dehiscence, the process of anther opening leading to pollen release, is crucial for pollination and thus reproduction in seed plants. Due to its importance, anther dehiscence has been investigated for many years (1–3). Already almost two centuries ago, Purkyně in his pioneering works described various anther tissues that are crucial for dehiscence in several species (4). Since then, a wide range of plants have been used for research, including many nonmodel species (5–7). In general, different types of tissues can be distinguished in angiosperm anthers: tapetum, middle layer, endothecium, epidermis, septum, and stomium (8, 9). Each tissue is essential and contributes to pollen and anther development or dehiscence. There are well-described developmental processes that precede dehiscence, such as tapetum degradation (10) or secondary cell wall thickening in the endothecium (11).

It has been proposed that dehiscence requires tissue dehydration (5, 12). Dehydration is facilitated by water transport within the anther (13, 14) and controlled transpiration via stomata (5). As transpiration rates depend on external conditions, the effect of high humidity has been investigated, assuming that it interferes with anther opening. However, these experiments on various species like rice (15), apricot or peach (16), and pecan (17), were performed on detached stamens or flowers, potentially creating artifacts. Although the impact of weather, particularly humidity, on dehiscence may offer plants a competitive advantage by selectively exposing pollen to pollinators (18, 19), the underlying mechanisms are not yet fully understood.

The water balance in plant tissues is generally regulated by alternation of stomata aperture, and anther dehydration is no exception. This is evident in the *Arabidopsis thaliana inducer of cfb expression1-2* (*ice1-2*) mutant, which fails to open anthers due to disrupted stomatal development (20). In the context of transpiration, water transport is no less important. For example, tobacco (*Nicotiana tabacum*) plants with RNA-silenced aquaporin PIP2 exhibit delayed anther dehiscence, highlighting the relevance of controlled water movement (14). In *A. thaliana* anthers, water transport is regulated by jasmonic acid (JA) (21), as shown in mutants with disrupted JA biosynthesis *defective in anther dehiscence1* (*dad1*) and *delayed dehiscence1* (*dde1*) that display abnormal anther dehiscence due to lack of dehydration (22, 23).

Developmentally controlled programmed cell death (dPCD) of different tissues throughout anther development has been suggested as an important mechanism in anther development (24, 25). dPCD in the tapetum has been reported as an essential process for both pollen and anther development in rice (*Oryza sativa*) (26, 27) and *Arabidopsis* (28). In *N. tabacum*, genetic ablation of stomium and surrounding tissues inhibited dehiscence, implicating the necessity of timely and controlled stomium degeneration for anther dehiscence (29). Nonetheless, the coordination of cell death with anther dehydration remains unclear.

This study presents a comprehensive analysis of anther dehiscence in *A. thaliana*. Our controlled laboratory experiments provide evidence that ambient humidity and transpiration control anther opening. Genetic and cell biology studies revealed that dehiscence is promoted by a defined developmental program involving spatially and temporally controlled progression of dPCD in specific anther tissues.

## Results

### Anther Dehiscence Is Postponed by High Humidity.

**Fig. 1.**
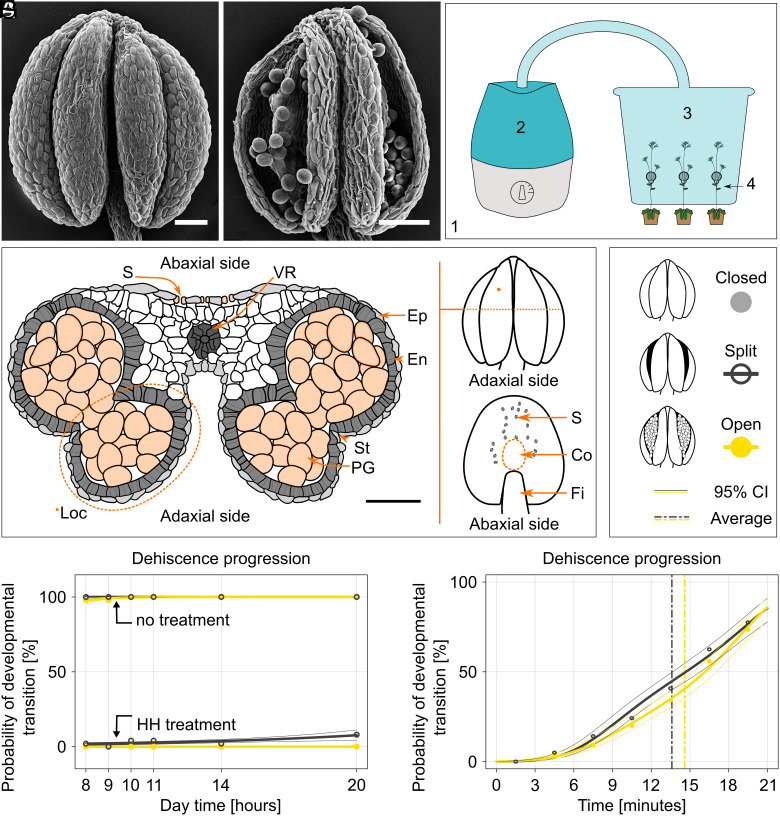
*A. thaliana* anthers do not open when exposed to HH in controlled conditions. (*A*) Closed *A. thaliana* anther. (*B*) Open *A. thaliana* anther. (*C*) Cultivation chamber (1) with HH generator (2) creating HH conditions in a box (3) with openings for stem placement (4). (*D*) Cross-section of a mature anther, *Loc. anther locule. *S*. stoma, *VR*. vascular region, *Ep*. epidermis, *En*. endothecium with secondary thickenings, *St*. stomium, *PG*. pollen grains. The scheme is based on the confocal z-stack of *A. thaliana* anther cross-section obtained with a vibratome. (*E*) Anther from the adaxial side; * indicates one of the four locules, and the dashed line indicates the cross-section. (*F*) Abaxial side of the anther; *S.* wild-type stomata density and distribution, *Co.* connective, *Fi.* filament. (*G*) Legend for the graphs (*H* and *I*) Light gray represents a closed anther at stage 12 according to Sanders et al. (8). Dark gray stands for anther with a narrow slit formed at the apex—stage 13. Yellow is for fully open anther at stage 14a. In graphs (*H* and *I*), coloured dots indicate the observed proportions of each developmental stage. Thick lines show the logistic regression model (GLM), with thin lines representing the 95% CI. (*H*) HH significantly prevents *A. thaliana* anthers from opening (*P* < 2.2e−16***, GLM logistic regression). 200 anthers were exposed to HH treatment from 5:00 to 20:00. Developmental stages were examined at 8:00, 9:00, 10:00, 11:00, 14:00, and 20:00. As a control in standard humidity, 200 anthers were analyzed at the same time. Legend in *G*. (*I*) Anther dehiscence was recorded over 21 min in 3-min steps after the HH treatment finished at 11:00 (120 anthers). The dashed lines indicate the average values. Figures *A* and *B* were obtained using SEM. The scale bar is 50 µm in all figures.

We investigated the hypothesis that ambient humidity affects dehiscence, which provides a potential link between weather conditions and pollen release. We established *A. thaliana* as a model system to examine the impact of high ambient humidity (HH) on anther dehiscence (Fig. 1 *A* and *B*), developing an experimental setup to control humidity under lab conditions (Fig. 1*C*). The primary advantage of this setup is the selective exposure to HH (100% relative humidity), exposing only inflorescence stems to HH while the rest of the plant remains at regular ambient humidity (AH, approx. 60%). Targeting specifically the inflorescences limits systemic effects and eliminates the need for detaching floral parts, leaving the examined plants intact.

Experiments always included only fully mature stamens of similar length as the pistil. The locules of mature anthers are surrounded by two cell layers, the epidermis and endothecium (Fig. 1*D*). The anthers of *A. thaliana* release pollen through two openings on the adaxial side, which are formed at the time of anther dehiscence (Fig. 1*E*). Of note, this adaxial side lacks stomata, while the abaxial side does form stomata (Fig. 1*F*). We designated three distinct categories to characterize anther dehiscence stages: closed (no visible opening), split (opening visible at the anther apex but no gaping), and fully open (pollen being released through gaping stomium) (stages 12, 13, and 14a, respectively, according to Sanders et al. (8), Fig. 1*G*). *A. thaliana* flowers were exposed to HH from 5:00 in the morning onward and dehiscence development was measured at 8:00, 9:00, 10:00, 11:00, 14:00, and 20:00. The HH-exposed anthers remained closed throughout the HH treatment (Fig. 1*H* and Dataset S1). Although at 20:00, 16 out of 200 anthers had split, none reached the fully open stage. In contrast, 196/200 anthers exposed to AH were fully open already by 8:00, the time when anther dehiscence occurs naturally. The remaining four anthers were split at 8:00 and fully opened 1 h later.

Next, we moved inflorescences to AH after 6 h of HH treatment, as our observations suggest that morning is the natural time window for anther dehiscence in *A. thaliana*. We then measured anther dehiscence progression in 3-min intervals for 21 min. Most of the analyzed 120 anthers split and fully opened between 14 and 15 min after exposure to AH (Fig. 1*I*).

As stamen/flower detachment has been widely used in previous studies, we also analyzed the effect of the detachment on dehiscence using the same experimental settings as described above. Contrary to intuition, detached flowers showed significant delays in anther dehiscence and full opening compared to attached flowers (*SI Appendix*, Fig. S1 *A*–*D*). This suggests that some dehiscence regulatory mechanisms, possibly involving stomatal function, require intact plants.

These experiments show that HH prevents anthers from splitting and full opening for a prolonged time and that dehiscence occurs within minutes after humidity decreases.

### Stomatal Density Affects Anther Dehiscence Dynamics.

**Fig. 2.**
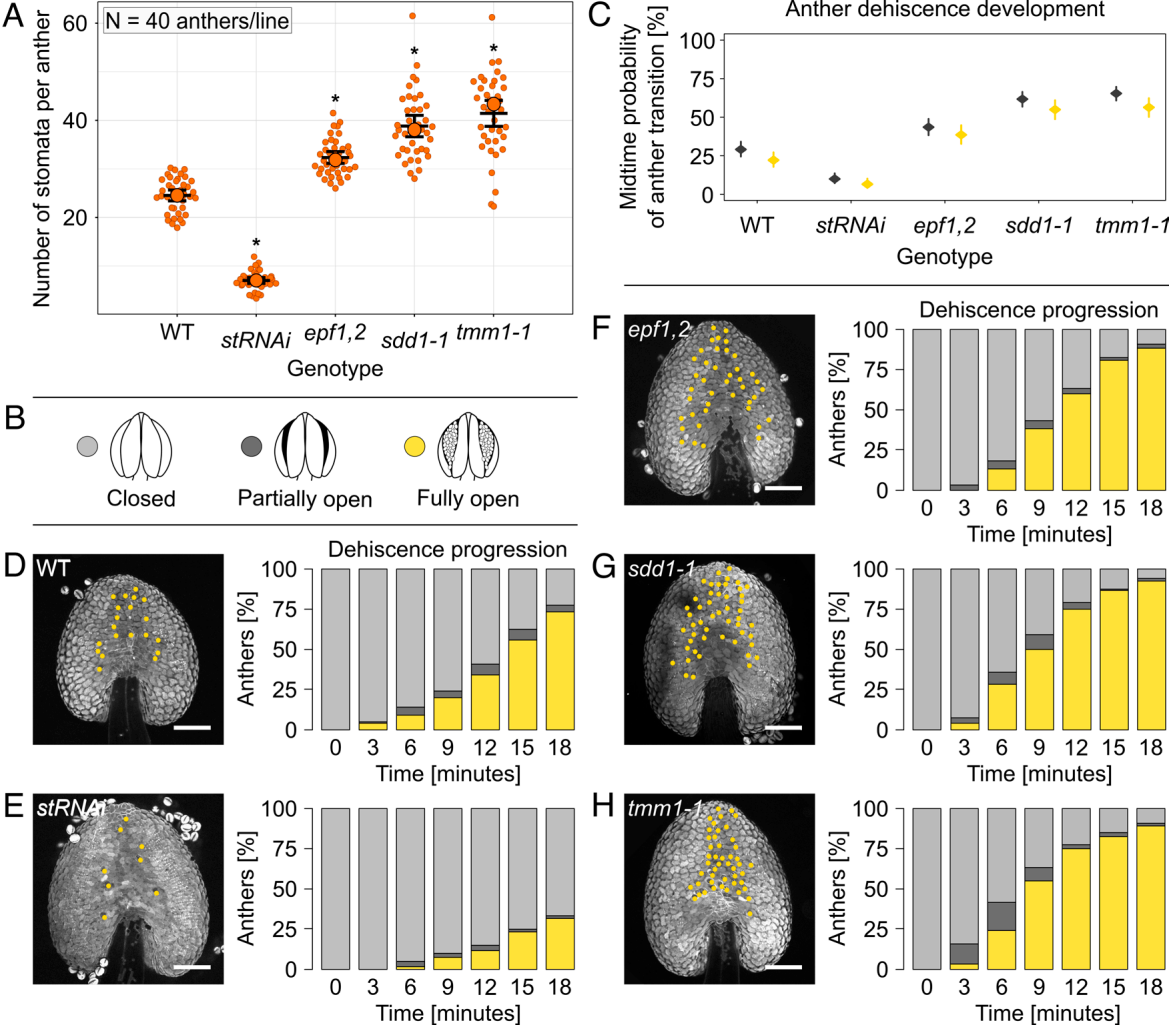
Anthers with higher stomata number and changed distribution (*epf1,2; sdd1-1; tmm1-1*) show accelerated opening compared to both wild-type (WT) and anthers with reduced stomata number (*stRNAi*). (*A*) Number of stomata per average WT anther area. The median (largest dots) and mean (widest horizontal lines) are shown, along with the ±bootstrap 95% CI. Significant difference to WT is indicated by asterisks. (*B*) Legend to the graphs C-H. Light gray represents closed anthers, dark gray partially open, and yellow fully open anthers with completely exposed pollen grains. (*C*–*H*) After high humidity treatment, anther dehiscence was measured over 21 min in 3-min intervals. In total, 120 anthers/line were analyzed. (*C*) Probability of anther splitting and opening in the midtime (10.5 min). In *stRNAi*, both anther splitting and opening rates are significantly reduced. In *epf1,2; sdd1-1; tmm1* splitting and opening rates increased (GLM, *P* < 2.2e−16***, rates and their 95% CI are presented). Legend in the figure. (*D*) WT anther: At 21 min, 27 anthers closed, 5 partially open, and 88 fully open. Legend in the figure applies on *D*–*H*. (*E*) *stRNAi* anther: At 21 min, 80 anthers closed, 2 partially open, and 38 fully open. (*F*) *epf1,2* anther: At 21 min, 11 anthers closed, 3 partially open, and 106 fully open. (*G*) *sdd1-1* anther: At 21 min, 7 anthers closed, 2 partially open and 111 fully open. (*H*) *tmm1-1* anther: At 21 min, 11 anthers closed, 2 partially open and 107 fully open. Legend: In figures *C*–*H*: light gray—closed anther—stage 12, dark gray—partially opened anther (splitting)—stage 13, yellow—fully opened anther stage 14a according to Sanders et al. (8). Abaxial sides of anthers are presented, each yellow dot represents a stoma. Anther visualization employed the autofluorescence of the cuticle using a blue channel on a spinning disc microscope, Zeiss Axio Observer.7/Yokogawa CSU-W1-T2. The scale bar is 100 µm in all figures.

As stomata control the gas exchange rate between the anther inner air spaces and the atmosphere and their dynamic aperture crucially determines transpiration rates, we tested whether stomatal density influences anther dehiscence, as had been previously suggested (20). We analyzed established *A. thaliana* mutant lines with higher stomatal densities [*epidermal patterning factor1,2* (*epf1,2*); *stomatal density and distribution1-1* (*sdd1-1*); and *too many mouths1-1* (*tmm1-1*), as well as a line silenced in *STOMAGEN-EPFL9* (*stRNAi*) with lower stomatal density (30, 31)]. We confirmed that anthers in *epf1,2*; *sdd1-1*; and *tmm1-1* exhibited higher stomatal numbers and altered spacing compared to the WT, whereas *stRNAi* showed lower stomatal numbers (Fig. 2*A* and *SI Appendix*, Fig. S2*A*). The size, shape, and developmental maturity of anthers were also assessed. Anthers of *sdd1-1*, *tmm1-*1, and WT were of comparable sizes, but anthers of *stRNAi* and *epf1,2* were on average larger than WT (*SI Appendix*, Fig. S2*B*). Both shape and developmental stage did not differ from the control (*SI Appendix*, Fig. S2*C*). Next, we exposed inflorescences to HH to prevent dehiscence and then moved them to AH conditions. The progression of anther dehiscence was measured over 21 min and scored as described above (Fig. 2*B*). Anthers with increased stomatal numbers, specifically *epf1,2*; *sdd1-1*; and *tmm1-1*, demonstrated significantly faster full opening compared to the WT, whereas *stRNAi* anther opening occurred slower (Fig. 2 *C*–*H*). Probabilities of transition from closed to split anthers were significantly higher for *epf1,2*; *sdd1-1*; and *tmm1-1* lines (43 ± 3%, 61 ± 3%, and 65 ± 2%, respectively) compared to the WT (29 ± 2%), whereas the splitting probability of *stRNAi* (10 ± 2%) was lower. Similarly, probabilities of full anther opening were significantly higher for mutant lines *epf1,2*; *sdd1-1*; and *tmm1-1* (38 ± 3%, 54 ± 3%, and 56 ± 3%) compared to WT (21 ± 2%) and much lower in *stRNAi* (6 ± 1%) (*SI Appendix*, Fig. S3 and Dataset S2).

The stomata role in anther dehiscence regulation can be further strengthened by evidence of the effect of abscisic acid (ABA), known for its ability to induce stomata closure (32). We demonstrated that ABA significantly delays anther dehiscence, as less than half of the ABA-treated WT anthers fully opened in AH, whereas nearly all control anthers did (*SI Appendix*, Fig. S4 and Dataset S3).

In conclusion, stomatal number significantly influences anther dehiscence duration, with higher stomatal densities resulting in faster opening and less stomata leading to slower opening. Although a difference in anther size for some of the used lines was noted, the changed dynamics of anther dehiscence cannot be explained by the difference in size alone. The crucial role of stomata in anther opening is further supported by the effect of ABA, which delays dehiscence.

### Endodermis and Endothecium Remain Fully Viable until Dehiscence Begins.

**Fig. 3.**
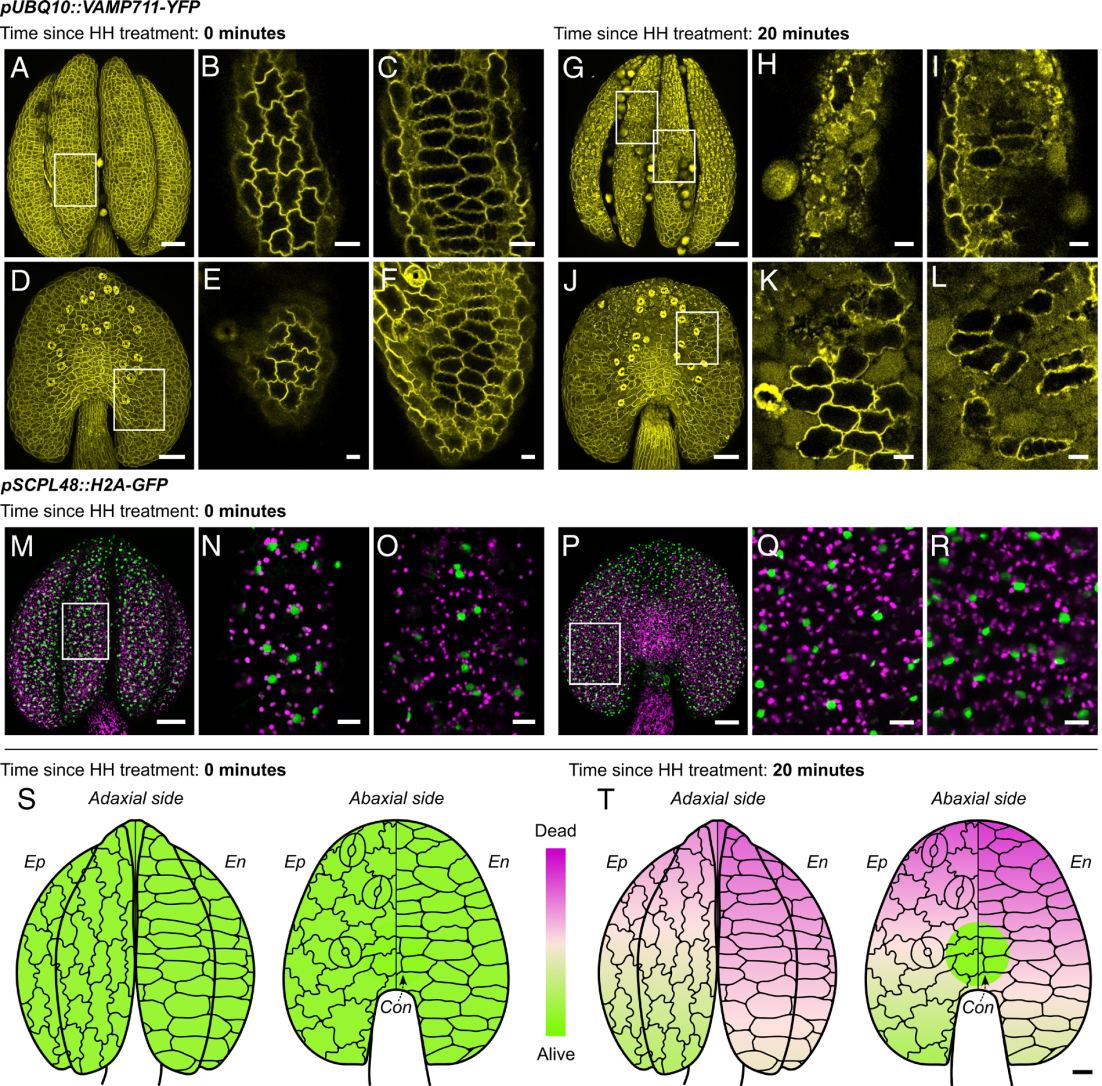
Anther tissues—epidermis, endothecium, and connective—are fully viable just before the opening starts. As the anther opens, the cells of mentioned anthers’ tissue undergo PCD in a direction-oriented manner. (*A*–*L*) *A. thaliana pUBQ10::VAMP711-YFP* anthers are closed until the opening starts when short (4 h) HH treatment is finished. Tonoplast degradation is visible 20 min into anther opening (tonoplast in yellow). Adaxial (AD, *A*) and abaxial side (AB, *D*) of closed anthers after short HH treatment, tonoplasts in both epidermis (*B* and *E*) and endothecium (*C* and *F*) are intact. AD (*G*) and AB side (*J*) of open anthers 20 min after short HH treatment, tonoplasts of the epidermis (*H* and *K*), and endothecium (*I* and *L*) are already degraded, but some cells remain intact. (*M*–*R*) *A. thaliana pSCPL48::H2A-GFP* anthers show cells that are predetermined to undergo PCD. GFP, shown in green, autofluorescence in magenta. The expression includes the whole anther tissue: AD (*M*) and AB (*P*) sides, epidermis (*N* and *Q*) and endothecium (*O* and *R*). (*S* and *T*) PCD progresses from the anther apex to the base. Epidermal cell’s lifespan is longer than the endothecial cells. Connective tissue stays viable for the last. In total, 112 anthers of these fluorescent marker lines were analyzed: *pHTR5::NLS-GFP-GUS*, *p35S::PIP2-GFP, pUBQ10::ToIM*, *pUBQ10::VAMP711-YFP*, *pCEP1::H2A-GFP*, *pDMP4::H2A-GFP,* and *pSCPL48::H2A-GFP*. Anthers were exposed to short HH treatment and observed 0 or 20 min after the HH treatment finished. Magenta represents completely dead cells, lawn green fully living cells, and mistyrose represents the transition between dead and living tissue. Each anther scheme has an epidermis (*Ep*) on the left side and an endothecium (*En*) on the right side. The cell schemes represent the assigned tissue of respected anther sides, AD and AB. *Con.* indicates connective tissue. The cells are approximately 4× bigger in comparison to anther size. The data were analyzed using beta regression in R (package betareg). (*S*) Fully intact short HH treated anthers at the time of finished treatment. Epidermis and endothecium are fully viable on both AD and AB sides. (*T*) Short-treated anthers 20 min after the treatment. PCD proceeds faster in the endothecium on both AD and AB sides. Anther apex contains more dead cells than the base. Connective is still viable. Legend: HH treatment—4 h. The figures were obtained using Leica TCS SP8. Scale bars are 50 µm in the anther overall view figures and 10 µm in close-up figures displaying cells. The scale bar for the anthers scheme is 10 µm and applies to the cells.

PCD has been implicated in anther development (24–26). Therefore, we assessed the viability of the different tissues in the dehiscing anthers using available *A. thaliana* marker lines for different subcellular compartments: *pHTR5::NLS-GFP-GUS* for nuclei (33), *p35S::PIP2-GFP* for the plasma membrane (34), *pUBQ10::VAMP711-YFP* for the tonoplast (35), and *pUBQ10::ToIM* for the integrity of the central vacuole (36). Inflorescences were exposed to HH for 4 h, stamens removed, and anthers imaged with a confocal microscope immediately (time 0), or 20 min after transfer from HH to AH. Both abaxial and adaxial sides were observed with the focus on the epidermis and endothecium. Anthers imaged at time 0 were fully closed and epidermis and endothecium cells were viable, indicated by intact cellular compartments (Fig. 3 *A*–*F* and *SI Appendix*, Figs. S5, S6, S7 *A*–*F*, and S9 *A* and *B*). 20 min after the exposure to AH, anthers were fully open and showed signs of cell death in both epidermis and endothecium (Fig. 3 *G*–*L* and *SI Appendix*, Fig. S9*E*), including vacuolar collapse (Fig. 3 *G*–*L* and *SI Appendix*, Figs. S7 and S8 *G*–*L*), nuclear envelope breakdown (*SI Appendix*, Fig. S5 *G*–*L*), and plasma membrane endodomain shedding (37) (*SI Appendix*, Fig. S6 *G*–*L*). Anther dehiscence time-lapses were also obtained confirming the above observations (*SI Appendix*, Fig. S10 and Movie S1). In addition, an intermediate stage was examined by analyzing anthers 10 min after transfer from HH to AH. At this stage, anthers had just begun to open, forming a narrow slit at the apex. This initial slit formation occurred prior to any detectable PCD in the epidermis or endothecium (*SI Appendix*, Fig. S9*C*). However, a further increase in the size of the slit was accompanied by PCD in both tissues (*SI Appendix*, Fig. S9*D*).

In summary, cells in the anther epidermis and endothecium are fully viable just before opening. Following initiation of anther opening, epidermal and endothecial cell death is not immediate, but the subsequent progression of anther opening correlates with the rapid onset of cell death in these tissues.

### Cell Death in Anthers Is a form of dPCD and Progresses in a Reproducible, Directional Pattern.

To characterize the cell death processes during dehiscence further, we examined promoter-reporter lines of dPCD-specific signature genes (*pCEP1::H2A-GFP*, *pDMP4::H2A-GFP*, and *pSCPL48::H2A-GFP*) (38). HH-treated plants were inspected as described above. At time 0 right after HH treatment, expression of nuclear-localized GFP of all dPCD-reporters was detected in the whole anther tissue, including the upper filament (Fig. 3 *M*–*R* and *SI Appendix*, Figs. S11, S12, and S13 *A*–*F*) suggesting a transcriptional preparation of dPCD in these tissues. Consistently, further imaging confirmed that the expression patterns remained unchanged under AH and various HH conditions, indicating that dPCD preparation occurs regardless of external humidity (*SI Appendix*, Fig. S14). Furthermore, signal diffusion indicative of chromatin degradation (39) was observed after 20 min in AH (*SI Appendix*, Figs. S11, S12, and S13 *G*–*L*). These results show that the rapid cell death observed in the epidermis and the endothecium during dehiscence has transcriptional parallels to established dPCD processes analyzed in other tissues, suggesting that this cell death process is a form of dPCD. This conclusion is supported by the analysis of several dPCD signature genes in publicly available RNA-seq data (*CEP1*, *DMP4*, *SCPL48* mentioned above and *BFN1*, *EXI1*, *PASPA3*, *RNS3*). Our analysis shows that the expression of all these genes, except *PASPA3*, is upregulated during final stages of anther development, further indicating that a canonical dPCD occurs during anther dehiscence (*SI Appendix*, Fig. S15 *A*–*G* and Datasets S4 and S5).

To analyze the precise spatial pattern of dPCD progression during dehiscence, we analyzed confocal z-stack images of several subcellular marker lines (*pHTR5::NLS-GFP-GUS*, *p35S::PIP2-GFP, pUBQ10::VAMP711-YFP*, *pUBQ10::ToIM*, *pCEP1/pDMP4/pSCPL48::H2A-GFP*; eight anthers imaged per line after 4 h of HH treatment, 10 and 20 min after shift to AH). Each anther area was divided into regions and scored based on viability status, ranging from completely intact cells to decompartmentalized cells (*SI Appendix*, Fig. S9*I*) in the epidermis and the endothecium. Data were then fitted using beta regression (Datasets S6 and S7). Our analyses revealed distinct differences among the tissues of closed, partially open, and fully open anthers. Closed anthers and anthers with only a narrow slit at the apex showed complete viability (Fig. 3*S* and *SI Appendix*, Fig. S9*C*), whereas partially open anthers with a wider slit and fully open anthers contained dead cells in both epidermis and endothecium (Fig. 3*T* and *SI Appendix*, Fig. S9*D*). By contrast, cells close to the connective area retained viability even after full anther opening. In both the epidermis and the endothecium, dPCD progression followed a basipetal pattern, with the anther base remaining viable the longest. Interestingly, we observed differences between the epidermis and endothecium: while the dPCD progression occurred in the same directional pattern in both tissues, dPCD in the endothecium spread faster toward the anther base than dPCD in the epidermis (Fig. 3*T*).

Taken together, the expression of dPCD signature genes, the rapid cellular decompartmentalization, and the distinctive pattern of cell death events suggest that a developmentally controlled, environmentally triggered dPCD process occurs during anther dehiscence.

### dPCD Manipulation Modulates Anther Dehiscence.

**Fig. 4.**
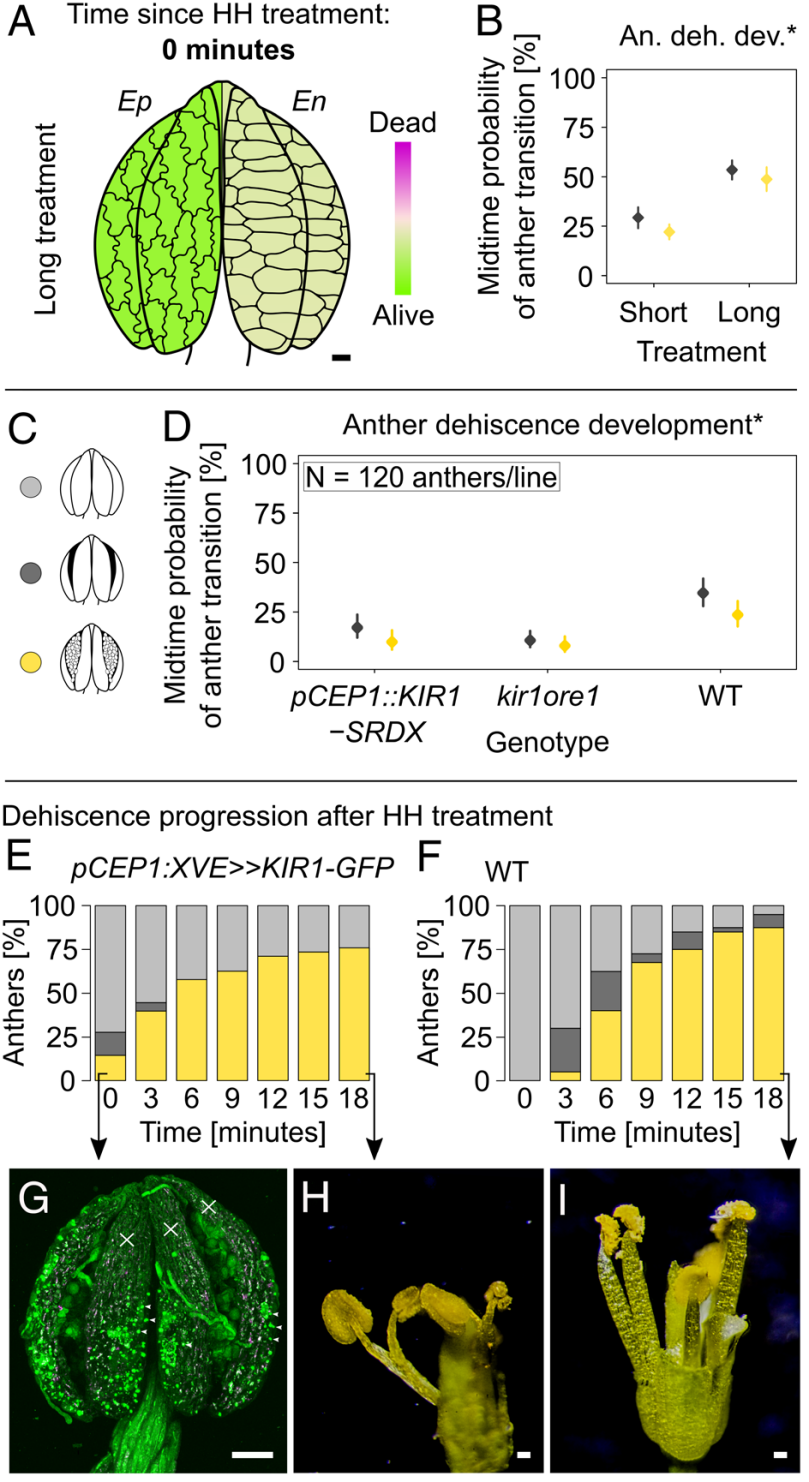
The PCD timing can be manipulated, resulting in changed anther dehiscence progression. Delayed PCD leads to slower opening. Earlier induced PCD execution results in opening despite high humidity treatment. (*A* and *B*) Anthers were exposed to long HH treatment (28 h). (*A*) Long-HH-treated anthers of listed lines were imaged and analyzed at the time of finished treatment: *pHTR5::NLS-GFP-GUS*, *p35S::PIP2-GFP, pUBQ10::ToIM*, *pUBQ10::VAMP711-YFP,* and *pCEP1/pDMP4/pSCPL48::H2A-GFP*. The epidermis is still intact, but the endothecium undergoes PCD. Lawn green shows fully living tissue, and mistyrose represents tissue where PCD occurs but is not completely dead yet. The data were analyzed using beta regression in R (package betareg). (*B*) Anther dehiscence development (*). Dehiscence was measured over 21 min in WT short (4 h) and long HH-treated anthers. Midtime opening and splitting probability is higher in WT long-HH-treated anthers (120 anthers/line, *P* < 2.2e-16***, rates and their 95% CI are presented). (*C*) Legend for the graphs *B* and *D*–*F*. Light gray represents closed anthers, dark gray stands for partially open anthers, and yellow for fully open anther with pollen grains that are entirely exposed. (*D*) Anther dehiscence was measured over 21 min in lines with reported delayed PCD, *pCEP1::KIR1-SRDX,* and *kir1ore1*. Midtime opening and splitting probability is lower in both lines than in WT (GLM, *P* < 2.2e-16***, rates and their 95% CI are presented, a legend in the figure). (*E*–*I*) Anther dehiscence was measured over 21 min in *pCEP1::XVE≫KIR1-GFP* and WT, treated with β-estradiol 24 h before the measurement. (*E*) Bar plots show the ratio of closed, split, and open anthers. At the time 0, 23 out of 83 *pCEP1::XVE≫KIR1-GFP* anthers are already split or open despite short HH treatment. Legend in the figure. (*F*) All 40 WT anthers are fully closed at the time 0. (*G*) At the time 0, *pCEP1::XVE≫KIR1-GFP* anther is open despite the HH treatment. Areas of dead tissue are marked with crosses and nuclei are indicated by asterisks. (*H*) *pCEP1::XVE≫KIR1-GFP* flower after 21 min, anthers are open and filaments are shrunk. (*I*) WT flower after 21 min, anthers are open and filaments are fully turgid. Legend: Figure *F* was obtained with Leica TCS SP8, the scale bar here is 50 µm. Figures *H* and *I* are from a stereomicroscope STM 822 5410 with a Nikon D3200 camera, scale bars are 100 µm. In Figure *A*, the scale bar is 10 µm and applies to the cells that are approximately 4× times bigger than anther size.

Building on the dPCD observations during anther dehiscence, we hypothesized that dPCD promotes anther opening. We analyzed the link between dPCD and anther dehiscence by exposing the above-mentioned marker lines (*pHTR5::NLS-GFP-GUS*, *p35S:: PIP2-GFP, pUBQ10::VAMP711-YFP*, *pUBQ10::ToIM*, *pCEP1/pDMP4/pSCPL48::H2A-GFP*) to 28 h of HH (long treatment) before transferring them to AH. This artificially delayed anther dehiscence by 1 d. Confocal imaging immediately after this long treatment revealed that the endothecium contained cells that had undergone dPCD, while the entire epidermis layer remained viable (Fig. 4*A* and *SI Appendix*, Figs. S5–S7, S8 *M*–*Y*, S9*G*, S11, S12, and S13 *M*–*Y*). After 20 min in AH, dPCD occurred in both cell layers, although more rapidly in the endothecium (*SI Appendix*, Fig. S9*H*). Dehiscence in WT anthers exposed to long HH treatment showed a splitting and full-opening probability of 48 ± 3% and 56 ± 3%, respectively, 10.5 min after the transfer from HH to AH. This probability is twice that of short-HH-treated (4 h) WT anthers after the transfer from HH to AH (Fig. 4 *B* and *C*, *SI Appendix*, Fig. S16 *A* and *B*, and Dataset S8), showing that extended HH treatment delays, but does not altogether block dPCD in anther tissues, correlating with anther dehiscence dynamics under these conditions.

To further test our hypothesis, we employed different approaches to either delay or induce dPCD by manipulating two established dPCD-promoting NAC transcription factors, *KIRA1* (*KIR1*) and *ORESARA1* (*ORE1*) (40), which we selected based on their expression in later stages of anther development, as seen in publicly available expression data (*SI Appendix*, Fig. S15 *H* and *I*).

First, we measured dehiscence after exposure to HH followed by transfer to AH in mutant lines that were shown to also affect stigmatic papilla lifespan (40). Both the *kir1ore1* double mutant as well as dominant-negative *pCEP1::KIR1-SRDX* lines exhibited a significant delay in dehiscence. At 10.5 min after removal from the HH chamber, the WT full opening probability was 27 ± 3%, compared to 8 ± 2% in *kir1ore1* and 10 ± 2% in *pCEP1::KIR1-SRDX* (Fig. 4 *C* and *D*, *SI Appendix*, Fig. S17 *A*–*D*, and Dataset S9). This identified a role of NAC-dependent dPCD gene regulatory networks during anther dehiscence and suggested that dPCD progression is required for fast anther opening after exposure to AH.

In a complementary approach, we induced precocious dPCD using an established estradiol-inducible overexpression of KIR1 in *pCEP1::XVE≫KIR1-GFP* line (*SI Appendix*, Fig. S18) (40). First, by analyzing *pCEP1::H2A-GFP* anthers, we confirmed the necessary activity of *CEP1* promotor 1 d before anthesis (*SI Appendix*, Fig. S17 *E*–*J*). Next, the *pCEP1::XVE≫KIR1-GFP* anthers were treated with an estradiol solution at stage 11 exactly 24 h before the anticipated dehiscence (*SI Appendix*, Fig. S17*K*). Anthers were then exposed to HH before transfer to AH as described above. Whereas the overall progress of dehiscence after the transfer to AH did not significantly differ between the induced and control variants (*SI Appendix*, Fig. S17 *S* and *T* and Dataset S9), a significant proportion of induced anthers fully opened even under HH conditions, forming slits from apex to base, while control anthers remained closed in HH (Fig. 4 *C*, *E*, and *F*). Induced *pCEP1::XVE≫KIR1-GFP* anthers, that fully opened despite HH, contained partially dead tissue, especially at the anther apex (Fig. 4*G* and *SI Appendix*, Fig. S17 *O*–*R*). Moreover, induced pCEP*1::XVE≫KIR1-GFP* filaments were visually shriveled (Fig. 4*H*), while control filaments appeared macroscopically turgid and viable at anther dehiscence (Fig. 4*I*), confirming the efficiency of the treatment to induce dPCD. These observations show that dPCD induction can overcome the inhibitory effect of HH on dehiscence and lead to anther opening even under HH conditions. However, dPCD alone seems not sufficient to enable the full opening and release of pollen grains, which necessitates AH to promote effective tissue dehydration.

In conclusion, by employing three different approaches, we demonstrate that dPCD processes in the anther epidermis and endothecium tightly correlate with anther dehiscence and that dPCD manipulation modulates dehiscence dynamics, strongly suggesting that dPCD processes in the anther epidermis and endothecium are a central part of an effective anther dehiscence mechanism.

## Discussion

### Environmental Humidity Regulates Dehiscence and Pollen Release.

Anther dehiscence is a process that occurs at the end of anther development and is crucial for timely and effective pollen release. It has been reasoned that it would be beneficial to reproductive success if pollen would be displayed to pollinators when they are most active and for many plants that would be during the day and under dry weather conditions (18). Dehiscence results from dehydration of anther tissue, presumably facilitated by aquaporin-promoted water transport within the anther tissue (14) and controlled transpiration through stomata (5, 25).

These observations propose that anther opening is not a passive dehydration process, but closely controlled by the plant in response to ambient conditions. Therefore, we investigated the effect of this weather parameter, mimicked by high humidity in strictly controlled laboratory conditions on intact plants and flowers. We show that high ambient humidity strongly affects anther opening, postponing both splitting and full opening. A drop in humidity triggers rapid anther dehiscence as the anthers fully open within 15 min after exposure to lowered ambient humidity. During this time, dehydration of the epidermis changes its mechanical properties resulting in complete anthers’ shape change (41).

Our conclusions are supported by a diversity of earlier observations from plant species such as rice (15), apricot, peach, almond (16), and pecan (17). These were, however, conducted on dissected flowers or stamens and we observed that dissection of flowers led to a significantly delayed anther dehiscence compared to dehiscence in anthers of intact plants. As a drought stress response may be triggered in the detached plant organs, it is important to consider another factor—phytohormones, as it is tempting to speculate that the drought-induced plant hormone ABA may negatively influence anther opening by causing stomata to close. In line with this, we showed that the application of ABA significantly delayed anther dehiscence under regular ambient humidity, as the opening of the ABA-treated anthers was considerably limited, while control anthers almost all opened. Anther opening is also dependent on JA, as mutation in the JA biosynthesis causes irregular dehiscence and hence male sterility (21). This is presumably due to disruption of anther dehydration which seems to be controlled by JA (21–23). Our observations strongly support the notion that plants can actively control dehiscence, as also suggested by dynamic changes in phytohormone levels observed in *Solanum lycopersicum* (42). Importantly, our results on *Arabidopsis* flowers reveal that anther opening in response to lowered ambient humidity can be a very rapid process, which necessitates analyses with high temporal resolution to dissect the regulatory mechanisms involved.

To verify the effect of humidity and transpiration on dehiscence, genetically, we examined various stomata mutants. Mutants with higher stomata number, *epf1,2*; *sdd1-1*; and *tmm1-1,* showed faster dehiscence, whereas *stRNAi* anthers with the least number of stomata displayed the most delayed dehiscence. This is in line with the earlier observations that *A. thaliana ice1-2* anthers exhibit disrupted dehiscence correlating with defective stomata development (20).

In summary, our controlled selective humidity treatments of intact flowers revealed that dehiscence is strongly delayed in high ambient humidity conditions and rapidly triggered by humidity decrease. Requirement of functional stomata further confirms that transpiration is a necessary part of a controlled anther dehiscence mechanism. These findings make it tempting to speculate that the stomata formed on the abaxial side of anthers are primarily important to facilitate a rapid tissue dehydration leading to anther opening.

### Anther Dehiscence Is Developmental Program Dependent on dPCD.

Previous studies have shown the involvement of dPCD in specific aspects of anther development, for instance, tapetum maturation (27). Also the degeneration of stomium cells during anther development has been observed and implicated in anther dehiscence as experiments suggest that dPCD processes in this tissue lead to gaping stomium (43). Our investigations revealed that in addition to controlled transpiration and water transport, also a rapidly triggered dPCD process in the epidermis and endothecium plays a role in effective and rapid anther opening.

Analyses of various fluorescent marker lines revealed that the anthers stayed closed, and epidermis and endothecium cells remained viable under high humidity conditions, supporting observations in the anther tissue in lily (44). After transfer to regular ambient humidity, anthers began to open by forming a narrow slit at the apex. The formation of this slit was not associated with dPCD in the epidermis and endothecium, but the widening of the slit to a full opening was correlated with a rapid progression of cell death in a basipetal fashion from the anther apex, with dPCD in the endothecium progressing most rapidly. Dying cells displayed features of cellular decompartmentalization typical for dPCD, with nuclei and vacuoles collapsing, and the plasma membrane exhibiting endodomain shedding (37). In addition, we detected the expression of dPCD signature genes (38), as well as established transcription factors regulating dPCD gene regulatory networks (39, 40) in predehiscence anthers. The subsequent evaluation of all marker lines showed a specific spatial pattern of dPCD progression in anthers.

We used genetic manipulation of dPCD to examine its importance for anther dehiscence using lines with delayed dPCD, *kir1ore1,* and *pCEP1::KIR1-SRDX* (40). Both lines exhibited delayed dehiscence compared to the WT. In a complementary approach, the induction of the precocious dPCD in the *pCEP1::XVE≫KIR1-GFP* revealed that dPCD is sufficient to promote dehiscence. Anthers with induced dPCD showed fully opened slit along the whole axis and partially exposed pollen grains even in high humidity. This shows that dPCD is required and sufficient for dehiscence; specifically, for the anther splitting.

As cells undergo dPCD, they shrink (45) and even small changes in turgor can induce large movements, depending on tissue characteristics (46). However, the overall shape of anthers was still maintained after precocious dPCD had been induced under high ambient humidity, supporting the necessity of transpiration and evaporation in addition to dPCD to achieve efficient and rapid anther dehiscence. According to a model based on lily and *Arabidopsis*, dehydration of the epidermis, together with secondary endothecial thickening, are the driving mechanical forces that facilitate the movement of the anther tissues from closed to open (41). Our analysis shows that dPCD in the epidermis and endothecium is sufficient for partial anther opening under high ambient humidity conditions, and necessary for rapid anther opening under regular ambient humidity conditions. Therefore, dPCD may on one hand accelerate dehydration processes and on the other contribute to tissue deformation through rapid loss of turgor pressure after cell death.

To conclude, anther dehiscence is a tightly regulated process that integrates a developmentally prepared PCD program with a rapid response to ambient humidity, providing a mechanism to optimize pollen release. As transcriptional preparation for dPCD in the epidermis and endothecium appears to be an integral part of anther maturation, a rapid onset of dPCD execution could be the posttranslationally triggered by a drop of ambient humidity. The underlying mechanism of the dPCD regulation by humidity, as well as control of stomata aperture to facilitate tissue dehydration are intriguing topics for future investigations.

## Materials and Methods

### Plant Material and Growth Conditions.

For this study, we used the following wild-type line: *A. thaliana* Col-0 ecotype; *A. thaliana* fluorescent marker lines used were *p35S::PIP2-GFP* (34), *pHTR5::NLS-GFP-GUS* (33), *pUBQ10::ToIM* (36), and *pUBQ10::VAMP711-YFP* (35). PCD-promoter marker lines used were *pCEP1::H2A-GFP*, *pDMP4::H2A-GFP,* and *pSCPL48::H2A-GFP* (38). Other PCD-related lines were employed: *kir1ore1*, *pCEP1::KIR1-SRDX,* and *pCEP1::XVE≫KIR1-GFP* (40). Stomata mutants used were *epf1,2*; *tmm1-1*; *stRNAi* (30) and *sdd1-1* (31). All the lines are listed in Dataset S10.

The seeds were sown on peat pellets (Jiffy). Fertilizer Kristalon Gold was used for the first watering (1 g of fertilizer per 1 L of water). Pellets with seeds were placed at dark and 4 °C for 7 d. Plants were then grown in long-day conditions (16 h light and 23 °C/8 h darkness and 18 °C; light intensity was 120 μmol m^−2^ s^−1^).

### High Humidity Treatment and Anther Dehiscence Measurement.

High humidity conditions were generated by the Lucky Reptile SuperFog II dew generator. This machine was connected through a plastic hose with a transparent plastic box (38 × 28 × 28 cm, Ikea). The box was closed by a lid which had an opening in it to put the hose which led the wet air from the machine to the box (Fig. 1*C*). Number of bigger and smaller holes were made in all the box sides at the height of grown *A. thaliana* plants. Through the bigger hole, a stem with the closed buds was placed inside and then moved to the smaller one down below. The bigger hole was then secured with a foam plug to reduce the high humidity leakage. In the evening prior to the experiment, old flowers were removed from the inflorescence, which was then placed inside the box. Treatment started at 5:00 and finished according to the experimental setup. To monitor the temperature and humidity in the HH box, an automatic system based on Raspberry Pi Zero W was developed using one Adafruit TSL2591 light sensor and two Adafruit BME280 temperature/humidity sensors placed in and out of the HH box. The relative air humidity inside the box during the treatment was 100%. The fully saturated air led to water condensation on the plant surface. The ambient humidity outside the box was approximately 60%. The stage of anther dehiscence was recorded once per hour and placed back into the box (Fig. 1*H*) or the stems were removed from the box and the anther dehiscence progress was measured for 21 min in 3-min steps (Figs. I*I*, 2 *C*–*H*, 4 *B* and *D*–*F* and *SI Appendix*, Figs. S1 *B*–*D*, S3, S16 *A* and *B*, S17 *A*–*D*, *S*, and *T*) using a binocular stereomicroscope. The stages of anther opening were scored according to Sanders et al. (8): anthers closed—stage 12; anthers partially open—stage 13; anthers open—stage 14a (Fig. 1*G*), the ambient temperature was measured by a mercury thermometer.

### Gene Expression Induction.

The gene induction was based on Gao et al. (40) protocol. For this experiment, flower buds 1 d before anthesis were chosen. At this developmental stage, anthers were already yellow and reached more than half of the pistil size (*SI Appendix*, Fig. S17*K*). All the sepals and petals were removed from the flower together with a pistil. All unmatured buds were also removed from the stem. The stem was then snapped to a wooden skewer and the flower was placed in an upside-down PCR tube also snapped to the skewer. The tube was filled with 350 µL of a working solution containing 1/20 MS and 100 µM β-estradiol in Dimethyl Sulfoxide (β-estradiol DMSO-based stock was 20 mM). The treatment lasted 6 h and was done 1 d before anthesis exactly 24 h before the anther opening measurement.

### ABA Treatment.

*A. thaliana* WT plants were exposed to high humidity from 5:00 to 9:00 to synchronize anther dehiscence and closure. After transfer to ambient humidity, freshly opened flowers were stripped of sepals, petals, and carpels, leaving only stamens attached to the plant. Stamens with closed anthers were treated for 2 h in either ABA solution (100 μM ABA in H_2_O, stock solution 50 mM in DMSO) or control solution (H_2_O with DMSO). Both solutions were adjusted to pH 6.5 with HCl or NaOH. After treatment, residual solution was absorbed with hydrophilic cellulose gauze. Anther dehiscence was scored 21 min later, as per Fig. 1*G*. The significance of ABA treatment was tested using a chi-squared test after fitting a generalized linear mixed effects model (**glmer** in R), with replication on different days included as a random effect. The model was applied individually to the measured proportions of closed, partially open, and fully open anthers.

### Cross-Sectioning and Staining.

Cross-sections were prepared to assess anther maturity in *A. thaliana* stomata mutants by observing anther wall cell layers. Flowers, with sepals, petals, and carpels removed, were embedded in 10% low-gelling agarose. The blocks were attached to a cutting base, placed in a water tank, and sectioned into 10 μm slices using a WPI Motorized Vibroslice NVSLM1. Sections, still in agarose, were stained with Calcofluor White (10 μL of 1 mg/mL stock in 1 mL H_2_O, Sigma) for 10 min, rinsed, and observed. To examine anther shape, flowers with only stamens remaining were treated with clearing solution (60 g chloral hydrate, 3.75 mL glycerol [both Sigma-Aldrich], 25 mL H_2_O) for 7 min, stained with 0.01% Auramine O (w/w, K. Hollborn & Söhne) for 5 min, rinsed, and observed.

### Confocal and Light Microscopy.

For confocal microscopy imaging, Stamens were attached to double-sided tape by their filaments and embedded in 2.5% low-gelling agarose (Sigma-Aldrich), and a covered slide was placed on top. All the figures (except for Figs. 1 *A* and *B* and 2 *D*–*H*) were obtained using a confocal microscope Leica TCS SP8 with the 20×/0.75 IMM objective using water immersion. The excitation and emission spectra were as follows: Auramine O λ_EX_ = 405 nm, λ_EM_ = 429 to 549; Calcofluor White λ_EX_ = 405 nm, λ_EM_ = 416 to 477 nm; chloroplasts autofluorescence 561 nm, λ_EM_ = 614 to 749 nm; GFP λ_EX_ = 488 nm, λ_EM_ = 495 to 540 nm or 495 to 550 nm; ToIM - GFP λ_EX_ = 488, λ_EM_ = 495 to 540 nm, tagRFP λ_EX_ = 561 nm, λ_EM_ = 570 to 660 nm; YFP λ_EX_ = 514 nm, λ_EM_ = 520 to 580 nm. The figures for Fig. 2 were obtained using a vertical stage (47) spinning disc microscope Zeiss Axio Observer.7/Yokogawa CSU-W1-T2 with a VS-HOM1000 excitation light homogenizer and with the use of Plan-Apochromat 20×/0.8 M27 objective. The autofluorescence of the cuticle was visualized using a blue channel, λ_EX_ = 405 nm. For time-lapse microscopy, plants were transferred to the microscopy room at 5:40 in the morning, avoiding light exposure. Under minimal illumination, the inflorescence, still attached to the plant, was secured to a microscope slide using double-sided tape. The target flower bud was carefully opened with tweezers, and petals and sepals were fixed to the tape. To improve visibility, the stigma and some pistils were removed. The slide and plant were placed on a vertical-stage spinning disk microscope for dry-mount imaging (λ_EX_: 488/561 nm for *pUBQ10::ToIM*, 515 nm for *pUBQ10::VAMP711-YFP*, Plan-Apochromat 10×/0.45 M27, z-step 7 to 10 μm, time step 30 to 60 s). Brightfield images were taken with a Nikon D3200 camera and STM 822 5410 stereomicroscope.

### Scanning Electron Microscopy.

Phosphate buffer (PBS, pH 7.2) was prepared for SEM sample preparation. Solution A (7.146 g Na_2_HPO_4_·12H_2_O in 100 mL) and Solution B (2.76 g NaH_2_PO_4_·H_2_O in 100 mL) were mixed to create a 0.2 M PBS stock (36 mL A + 14 mL B). Samples were fixed in 2.5% glutaraldehyde (Grade I, Sigma-Aldrich) in 0.1 M PBS for 24 h at 4 °C and then washed in 0.1 M PBS for 12 h at 4 °C and distilled water for 10 min at room temperature. Dehydration followed an EtOH series: 35% (15 min), 50% (15 min), 70% (30 min), 80% (15 min), 96% (15 min), and 100% (15 min). Samples, kept wet in 70% EtOH, were transferred to paper envelopes, dried in a Bal-Tec CPD 030 critical point dryer, coated with a 2 nm gold layer (Bal-Tec SCD 050), and observed using a JEOL JSM-IT200 SEM.

### Statistical Analysis of Anther Opening and Stomata Mutants.

Logistic regression of HH treatment experiments on *A. thaliana* was analyzed by Generalized Linear Model (GLM) with binomial distribution and individual effect significance was calculated by χ^2^-test. 95% CI were calculated by confint function with default setting. Anther opening after HH treatment was analyzed by logistic regression (GLM with binomial family of distribution). Variant identifiers, time, and its quadrate were used as predictors in a model with a full set of interactions. Optimal model was determined by a stepwise algorithm according to Akaike Information Criterion (AIC, step function) and tested by χ^2^-test (anova function). Differences between individual variants were estimated by emmeans function from package emmeans. The PlotsOfData web app (48) was used to plot the data and test the significance of differences in stomata number (Fig. 2*B*) and abaxial size (*SI Appendix*, Fig. S2*B*).

### Quantification of Cell Death Progression in Anthers.

PCD progression was analyzed in short (4 h, 5:00 to 9:00) and long (28 h, 5:00 d 1 to 9:00 d 2) HH-treated anthers from fluorescent marker lines *pHTR5::NLS-GFP-GUS*, *p35S::PIP2-GFP*, *pUBQ10::ToIM*, *pUBQ10::VAMP711-YFP*, *pCEP1::H2A-GFP*, *pDMP4::H2A-GFP,* and *pSCPL48::H2A-GFP*. Anthers were imaged immediately, 10, or 20 min posttreatment as dehiscence progressed. Dead cell proportions were assessed semiquantitatively (>10%, 10 to 40%, 40 to 60%, 60 to 90%, and >90%) across apical, central, and basal regions (plus connective, if applicable) for both adaxial and abaxial sides, scoring epidermis and endothecium separately (*SI Appendix*, Fig. S8*I*). The reliability of scoring was enhanced by horizontal averaging of corresponding areas. Spatial cell death patterns relative to dehiscence and HH duration were analyzed using beta regression in R (49) (packages **betareg** and **StepBeta**), with model selection via StepBeta and significance tested by likelihood ratio (lrtest, package **lmtest**). The full R script (CellDeathQuant.md) is available on GitHub (50). A total of 144 anthers were analyzed, with 370 anthers imaged across all lines and time points that were not included.

### Analysis of PCD-Related Gene Expression.

The set of RNA-seq libraries from 12 different stages of *A. thaliana* anther, three transcriptomes of mature pollen, and one transcriptome of filament was obtained from the ENA repository (in total 22, 10, and 2 libraries, Dataset S4) and quantified by Kallisto 0.48.0 using Araport11 representative CDS model as a reference (Dataset S5). For other libraries, nonstandardized TPM values of transcripts of interest were fitted by the GAM model [R package **mgcv** (51)], while medians were counted in the case of pollen and filament libraries.

### Image Analysis.

Brightfield image series were focus-stacked using PICOLAY (www.picolay.de) and adjusted in Zoner Photo Studio X (https://www.zoner.cz). All figures were processed in Fiji (52). For stomata number analysis, the Fiji macro *surface_.ijm* was used to generate surface figures. The abaxial size of stomatal mutant anthers was measured via thresholding to create binary images. Time-lapse imaging involved processing 16-bit z-stacks in Fiji, applying maximal projection, nonlinear brightness adjustment (RawTherapee 5.8, PP3 conversion files in Datasets S11 and S12), and converting to 8-bit AVI. Figures were assembled in Inkscape (53).

## Supplementary Material

Appendix 01 (PDF)

Dataset S01 (XLSX)

Dataset S02 (XLSX)

Dataset S03 (XLSX)

Dataset S04 (XLSX)

Dataset S05 (XLSX)

Dataset S06 (XLSX)

Dataset S07 (XLSX)

Dataset S08 (XLSX)

Dataset S09 (XLSX)

Dataset S10 (XLSX)

Dataset S11 (XLSX)

Dataset S12 (XLSX)

Movie S1. **Using a spinning disc microscope with a dry mounting approach, the progression of anther dehiscence can be recorded**. As the stamens remain attached to the plant throughout the process, this sample preparation allows for a fully intact observation. 20-min time lapse of *A. thaliana pUBQ10::VAMP711-YFP* anther dehiscence is shown, the tonoplast is in magenta-fire. All anthers are fully open at the end of this recording. Time is written mm:ss. The scale bar is 100 μm.

## Data Availability

All study data are included in the article and/or supporting information. Codes used in this study are deposited in a publicly accessible GitHub repository (50).
