# Supplementary material for: Hygrometrically controlled programmed cell death drives anther opening and pollen release
Source: Proc Natl Acad Sci U S A. 2025 May 16;122(20):e2420132122. doi: 10.1073/pnas.2420132122 (PMC12107150; doi:10.1073/pnas.2420132122)
Supplement: Supplementary file 1 — Appendix 01 (PDF) [file pnas.2420132122.sapp.pdf]

Supporting Information for

Hygrometrically controlled programmed cell death drives anther opening and pollen release

Anna Kampová[1]*, Moritz K. Nowack[2,3], Matyáš Fendrych[1], Stanislav Vosolsobě[1]*.

[1] Department of Experimental Plant Biology, Faculty of Science, Charles University, Prague, Czechia
[2] Department of Plant Biotechnology and Bioinformatics, Ghent University, Ghent, Belgium
[3] VIB Center of Plant Systems Biology, Ghent, Belgium

*Corresponding: Anna Kampová, Stanislav Vosolsobě.

Email: anna.kampova@natur.cuni.cz
stanislav.vosolsobe@natur.cuni.cz

This PDF file includes:

Figures S1 to S18
Legend for Movie S1
Legends for Datasets S1 to S12
SI References

Other supporting materials for this manuscript include the following:

Movie S1
Datasets S1 to S12

Supplementary figures S1–S18:

Detached and attached WT *A. thaliana* flowers

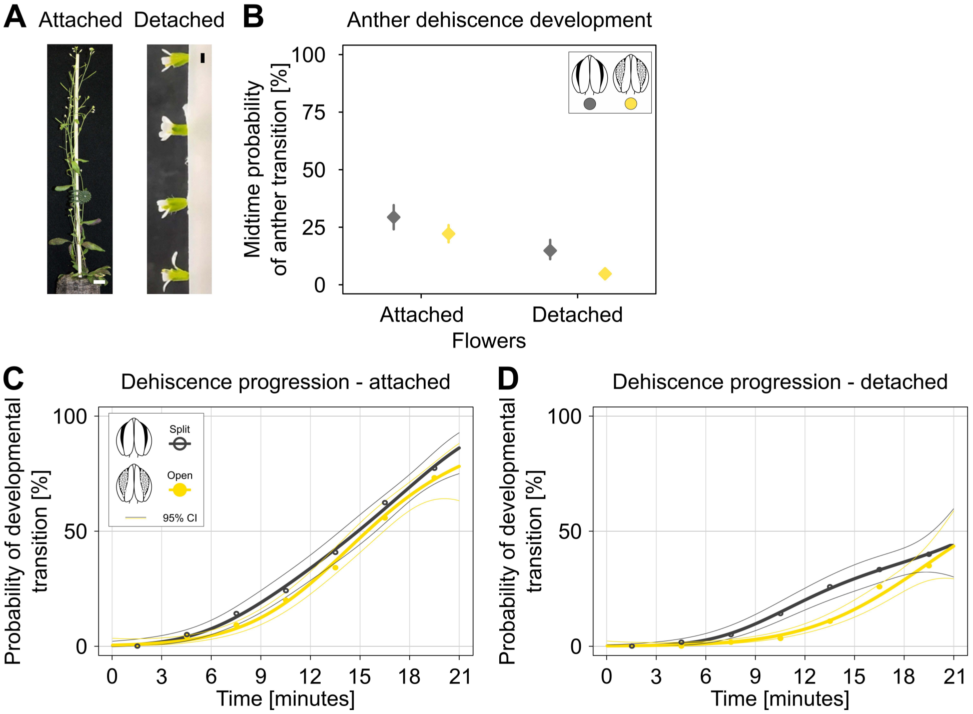

Fig. S1. Anther dehiscence is slower in *A. thaliana* flowers detached from plants. A. Left: Still attached flowers, scale bar is 1 cm. Right: Detached flowers, scale bar is 5 mm. **B-D.** The development of 120 anthers from both attached and detached flowers was monitored for 21 minutes in 3-minute steps after short high humidity (HH) treatment was completed. Flower detachment significantly reduced both anther splitting rate (partially open anther in dark grey) and opening rate (fully open anther in yellow; GLM, $P < 2.2e\text{-}16^{***}$, 95% CI are shown). **B.** Rates from half-time of the measured period (10.5 minutes) are shown. **C**. Dehiscence progression of attached anthers. **D.** Anther dehiscence progression of detached flowers. **Legend:** Coloured points in C. and D. show measured proportions of respective developmental stages, logistic regression (GLM) is indicated by thick lines with 95% CI – thin lines (legend shown in figure C).

A

WT and stomatal density/distribution mutants

Line	Stomata #	Description of phenotype
WT	24,5	no clusters or joined stomata
stRNAi	6,8	no clusters or joined stomata
epf1,2	32,3	clusters, joined stomata (2 stomata)
sdd1-1	38,8	clusters, joined stomata (2-4 stomata)
tmm1-1	41,4	clusters, joined stomata (2-7 stomata)

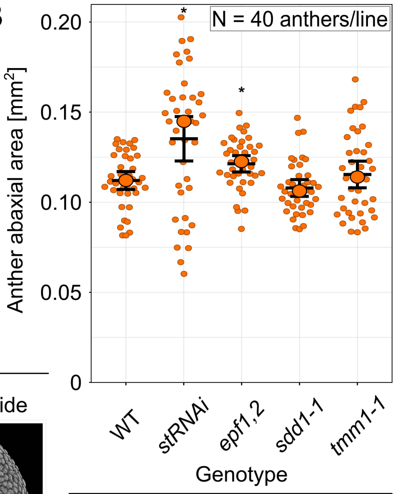

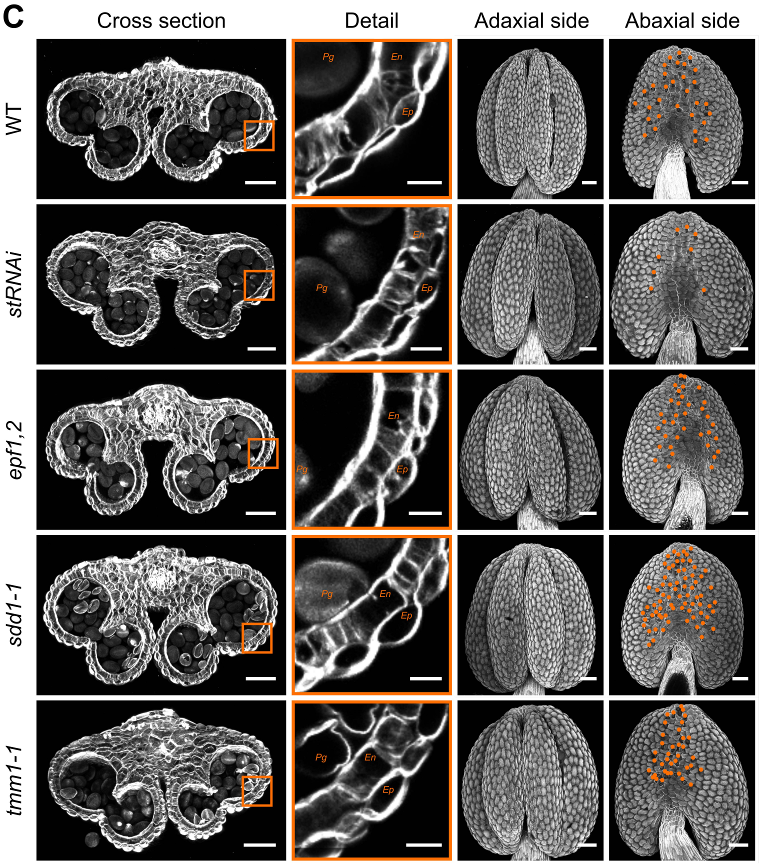

Fig. S2. Characterisation of *A. thaliana* wild-type (WT) and stomatal mutant lines with respect to anther size, maturity and shape. All lines differ in the number of stomata, but their shape and maturity are comparable. The anthers are also of a similar size, with the exception of *epf1-2* and *stRNAi,* which have generally larger anthers compared to the WT.

A. Table describing the stomatal mutants. **B.** Anther abaxial sizes of 40 anthers per line are shown (in mm^2). Anthers are larger in *epf1-2* and *stRNAi*, with the latter displaying the greatest variability in size. Medians (largest points) and means (broadest horizontal lines) are shown together with 95% confidence intervals of means. Significant differences from WT are indicated by asterisks. **C.** Cross-sections showing details of anther walls are presented with the whole anther figures from

adaxial and abaxial sides. Before the anther dehiscence initiation, the anther walls of all lines consist of only two layers, epidermis and endothecium. This indicates the anther maturity prior to anther dehiscence at stage 12 according to Sanders *et al.*, 1999(1). No diversity in the general shape of anthers was observed in any of the lines. **Legend:** *En.* endothecium, *Ep.* epidermis, *Pg.* pollen grains. Each point represents a stoma in abaxial side figures. Scale bars in cross section, adaxial and abaxial side figures are 50 μm. In detail figures the scale bars are 10 μm. The anthers were stained with Calcofluor White for cross sections and with Auramine O for total views. All the figures were obtained using confocal microscope Leica TCS SP8.

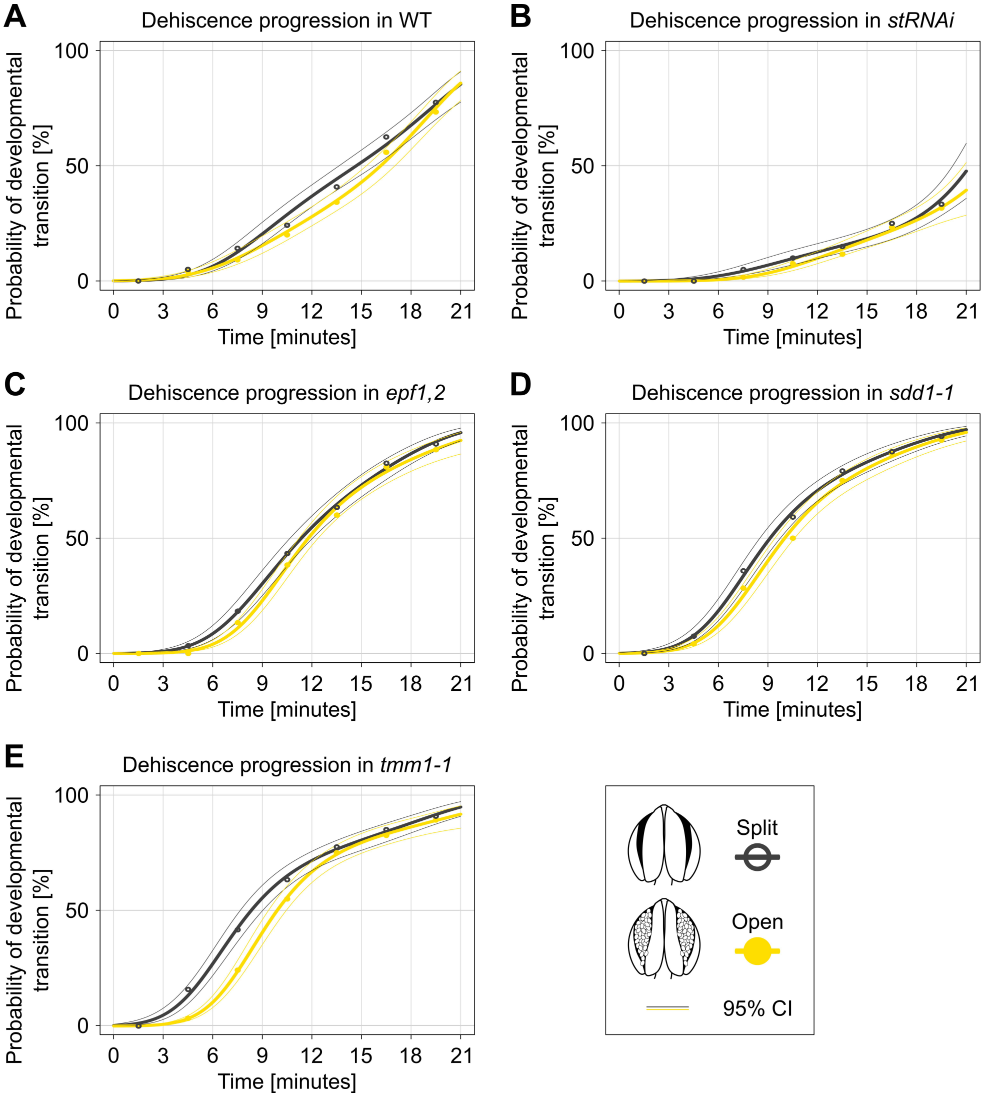

Fig. S3. Anthers of mutants with higher stomata density, *epf1,2; sdd1-1 and tmm1-1*, split and open significantly faster compared to WT and *stRNAi*, a line with reduced stomata number. The probabilities of splitting (dark grey) and opening (yellow) are displayed, showing the dehiscence process over a 21-minute timeframe in 3-minute steps. **A.** WT. **B.** *stRNAi*. Anther splitting and opening is notably decelerated. **C-E.** Anther splitting and opening are accelerated. **C.** *epf1,2*. **D.** *sdd1-1*. **E.** *tmm1-1*. **Legend:** The x-axis shows the time [minutes] and the y-axis indicates the probability rate of either splitting or opening. These graphs correspond with the bar plots from Figure 2. Coloured points show measured proportions of respective developmental stages, logistic regression (GLM) is indicated by thick lines with 95% CI – thin lines.

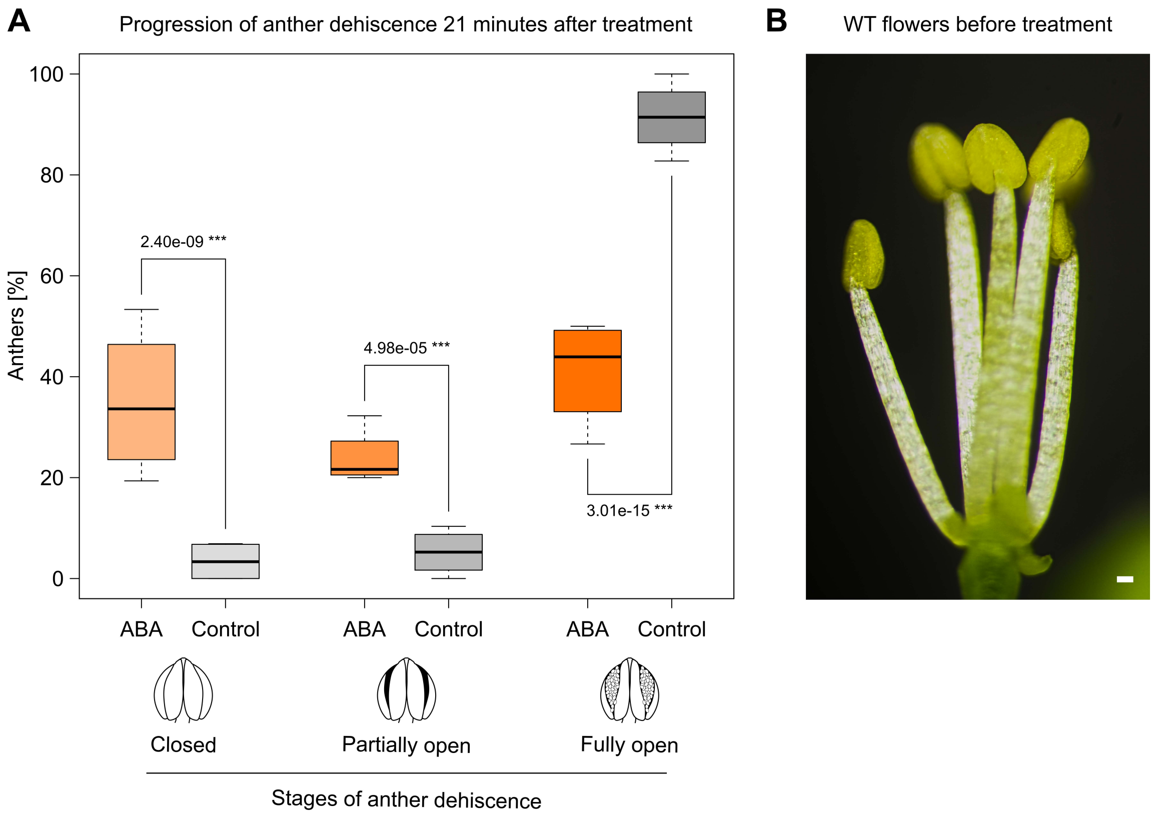

Fig. S4. Abscisic acid (ABA) significantly affects the dynamics of anther dehiscence in *A. thaliana*, specifically in terms of the speed of opening. A. Graph shows the progression of anther dehiscence 21 minutes after ABA and control treatments. Closed, pre-dehiscent anthers were treated with ABA (100 µM ABA in H_2O with ABA stock in DMSO, N = 102) or mock treatment (H_2O with DMSO, N = 111) for 2 hours, then exposed to regular ambient humidity (AH), and their opening was recorded 21 minutes later. Three stages of anther development are shown, closed, partially open, and fully open. In ABA-treated anthers, more than half are either closed or only partially open (34 and 25/102), and only 43/102 are fully open. In contrast, in the control group, most anthers are fully open (101/111), with a minority remaining closed or partially open (4 and 6/111). Statistical significance, determined by a generalized linear mixed-effects model, is indicated above/below the boxplots, which represent variability across treatment replications conducted on 4 different days. **B.** For effective treatments, flowers were stripped of sepals, petals, and carpel, but remained attached to the plant. The scale bar is 100 µm.

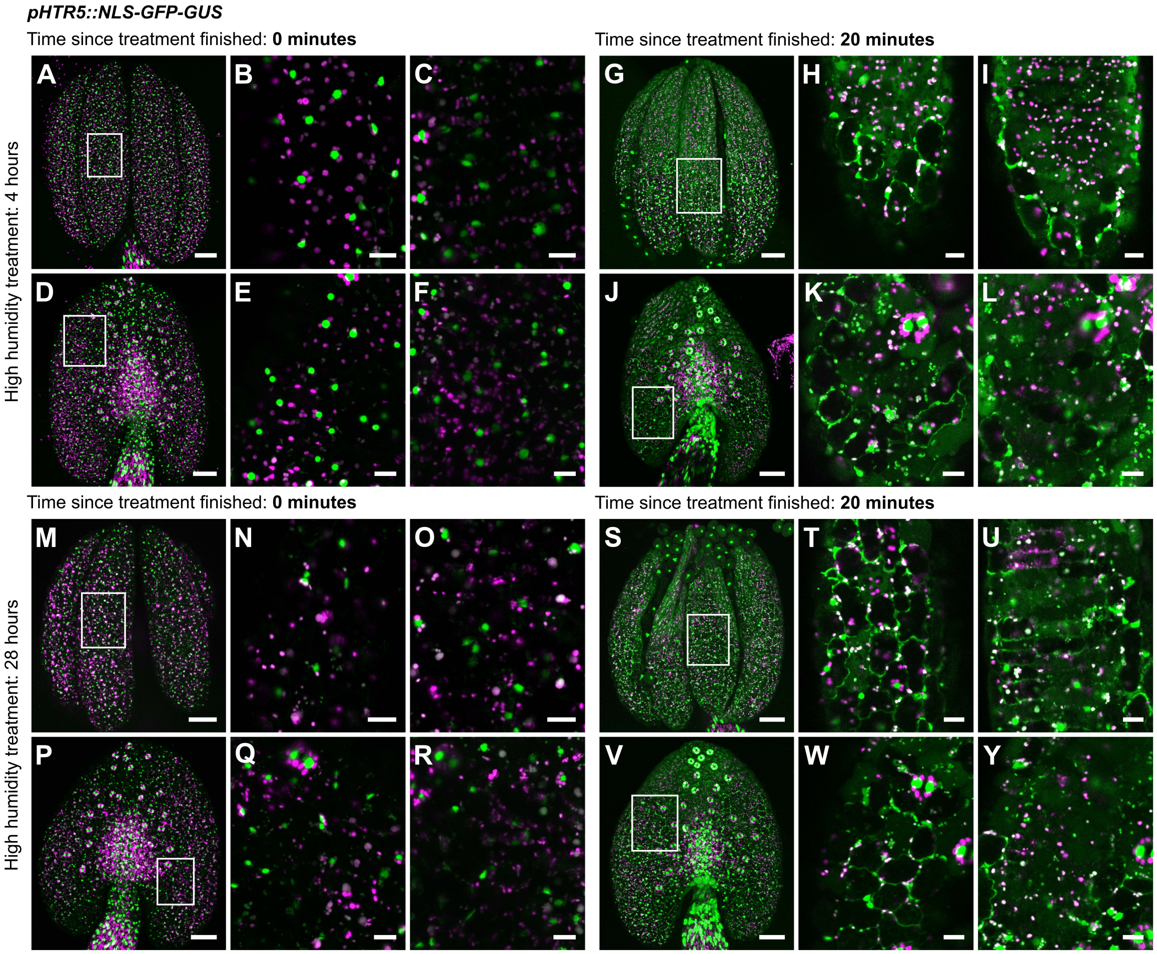

Fig. S5. *A. thaliana pHTR5::NLS-GFP-GUS* anthers stay closed when placed in HH conditions. Subsequent opening, when the treatment ends, is accompanied by programmed cell death (PCD). The PCD progresses from the apex to the base, stomata also undergo PCD in the same direction. Nuclei are shown in green, autofluorescence in magenta. Once open anthers partially regain their original shape when placed in a liquid medium. **A-F.** Closed short-treated anthers. Both epidermis and endothecium are intact. **G-L.** Open anthers after a short treatment. Nuclei collapse in both, the epidermis and endothecium. **M-R.** Closed long-treated anthers. Most of the cells are still intact. **S-Y.** Open anthers after a long treatment, PCD progresses in the direction towards the base, and both epidermis and endothecium are affected. **Legend:** Flowers were exposed to HH for 4 or 28 hours and imaged 0 or 20 minutes after transfer to AH. For each group of figures, the upper row represents the adaxial side, and the lower row represents the abaxial side. From left to right: overall figures, epidermis details, endothecium details. The figures were obtained using Leica TCS SP8. Scale bars are 50 μm in overall view figures and 10 μm in close-up figures.

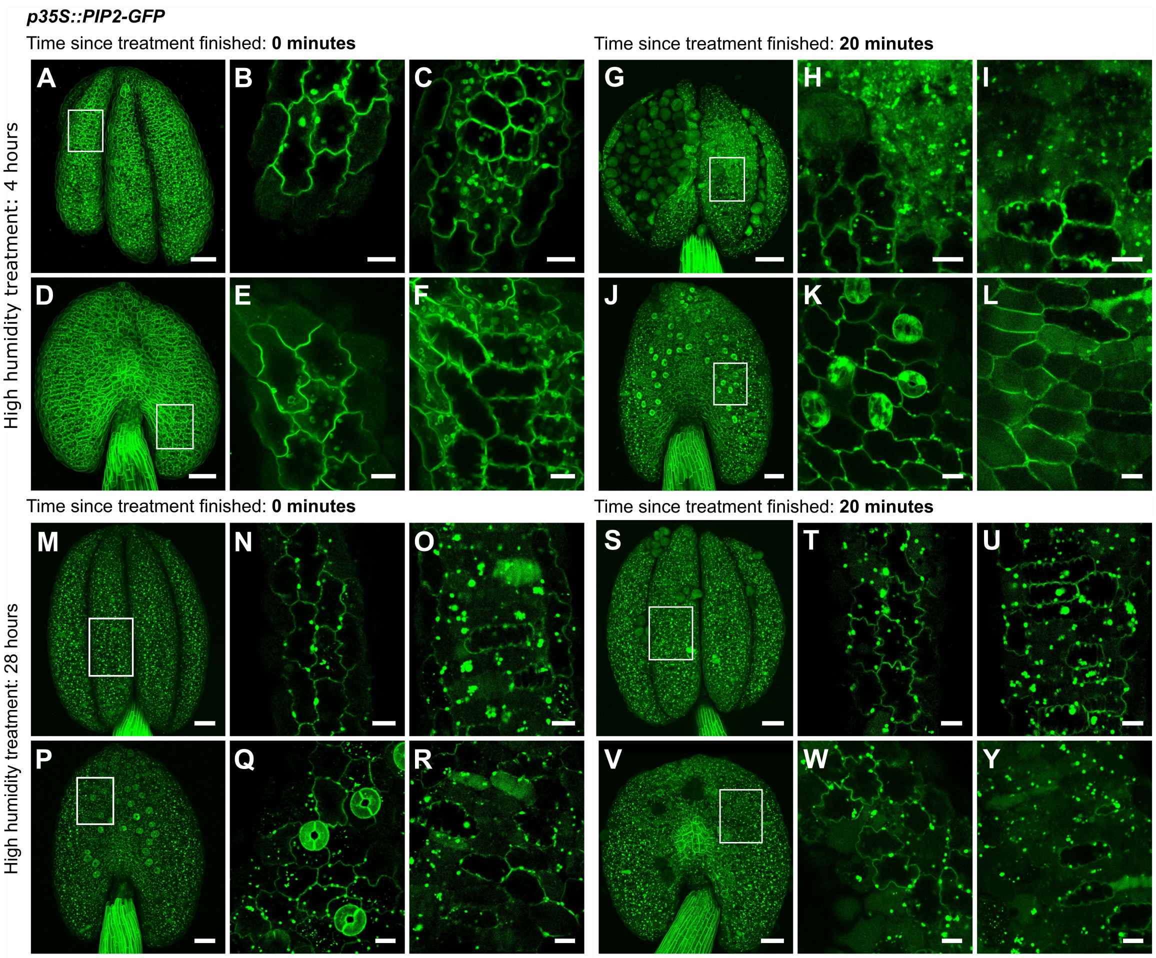

Fig. S6. Plasma membrane endodomain shedding is visible 20 minutes into anther opening. *A. thaliana p35S::PIP2-GFP* anthers remain closed until the opening initiation, induced by short and long HH treatment. After 20 minutes, changes related to PCD occur on the plasma membrane (PM, shown in green). While open anthers can partly regain their shape when embedded in 2,5% low melting agarose, the PM changes persist. **A-F.** Closed anthers after a short treatment, PM in both epidermis and endothecium remains intact. **G-L.** Open anthers after a short treatment, PM in both epidermis and endothecium is already degraded. **M-R.** Closed anthers after a long treatment, PM is intact in the epidermis but not in the endothecium. **S-Y.** Open anthers after a long treatment, PM in both epidermis and endothecium is visibly affected. **Legend:** Flowers were exposed to HH for 4 or 28 hours and imaged 0 or 20 minutes after transfer to AH. For each group of figures: the upper row represents the adaxial side, and the lower row represents the abaxial side. From left to right: overall figures, epidermis details, endothecium details. The figures were obtained using Leica TCS SP8. Scale bars are 50 µm in overall view figures and 10 µm in close-up figures.

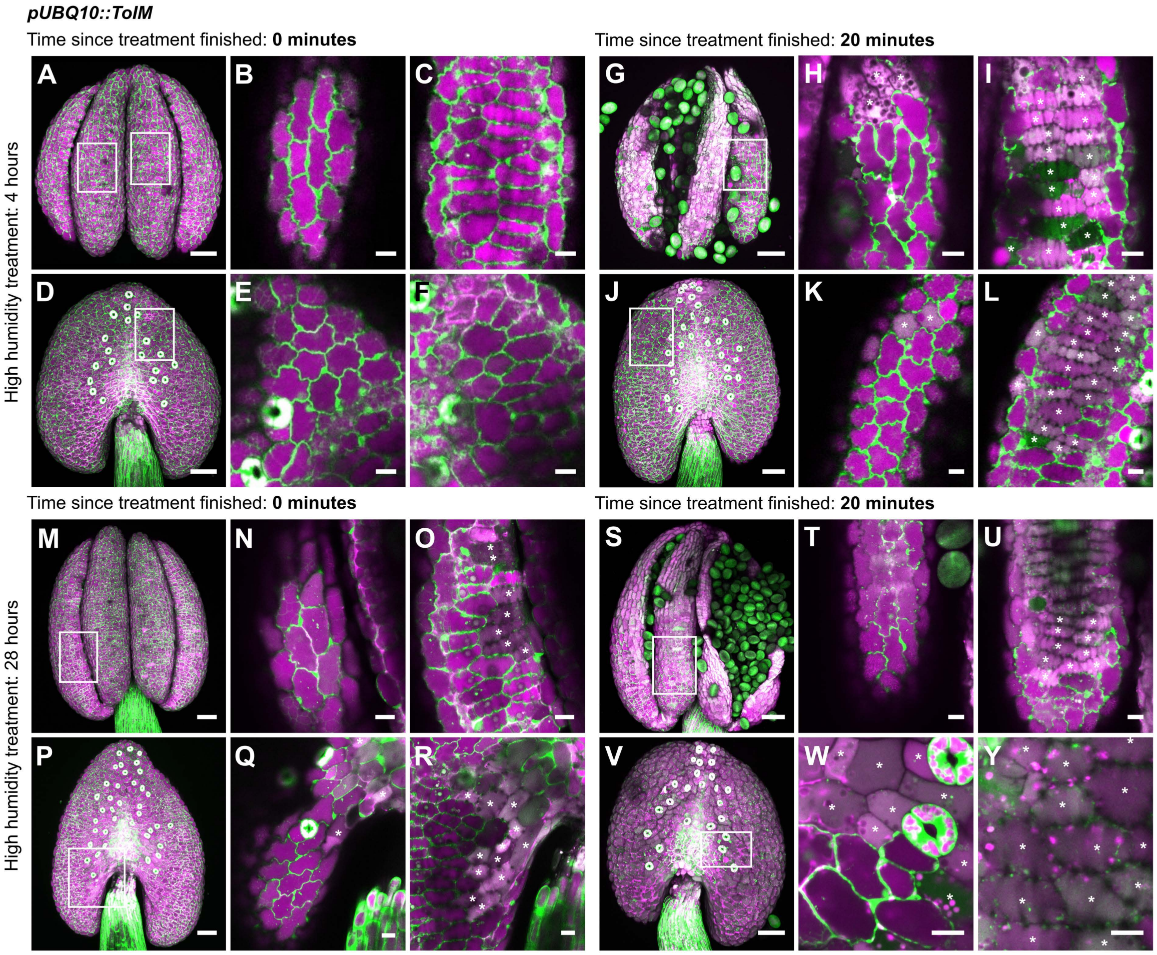

Fig. S7. The <u>To</u>noplast <u>I</u>ntegrity <u>M</u>arker (*A. thaliana pUBQ10::ToIM*) reveals progressing vacuole degradation within 20 minutes of anther opening. The vacuole is represented by magenta and the cytoplasm by green. Upon vacuole bursting, the signals merge, resulting in a light pink to white signal – such cells are marked with asterisks. Anthers remain closed until the completion of both short and long HH treatments. However, in closed long-treated anthers at the time 0, vacuoles undergo degradation in the endothecium. Next, the progression of vacuole degradation and signal merging is observed in open anthers of both treated variants. **A-F.** Short-treated anthers with intact epidermis and endothecium. **G-L.** Open anthers after a short treatment, showing visible vacuole degradation predominantly in the endothecium. **M-R.** Closed anthers after a long treatment, with vacuole degradation already occurring in the endothecium. **S-Y.** Open anthers after a long treatment, displaying vacuole degradation in both epidermis and endothecium. **Legend:** Flowers were exposed to HH for 4 or 28 hours and imaged 0 or 20 minutes after transfer to AH. For each group of figures, the upper row represents the adaxial side, and the lower row the abaxial side. From left to right: overall figures, epidermis details, endothecium details. The figures

were obtained using Leica TCS SP8. Scale bars are 50 μm in overall view figures, 10 μm in close-up figures.

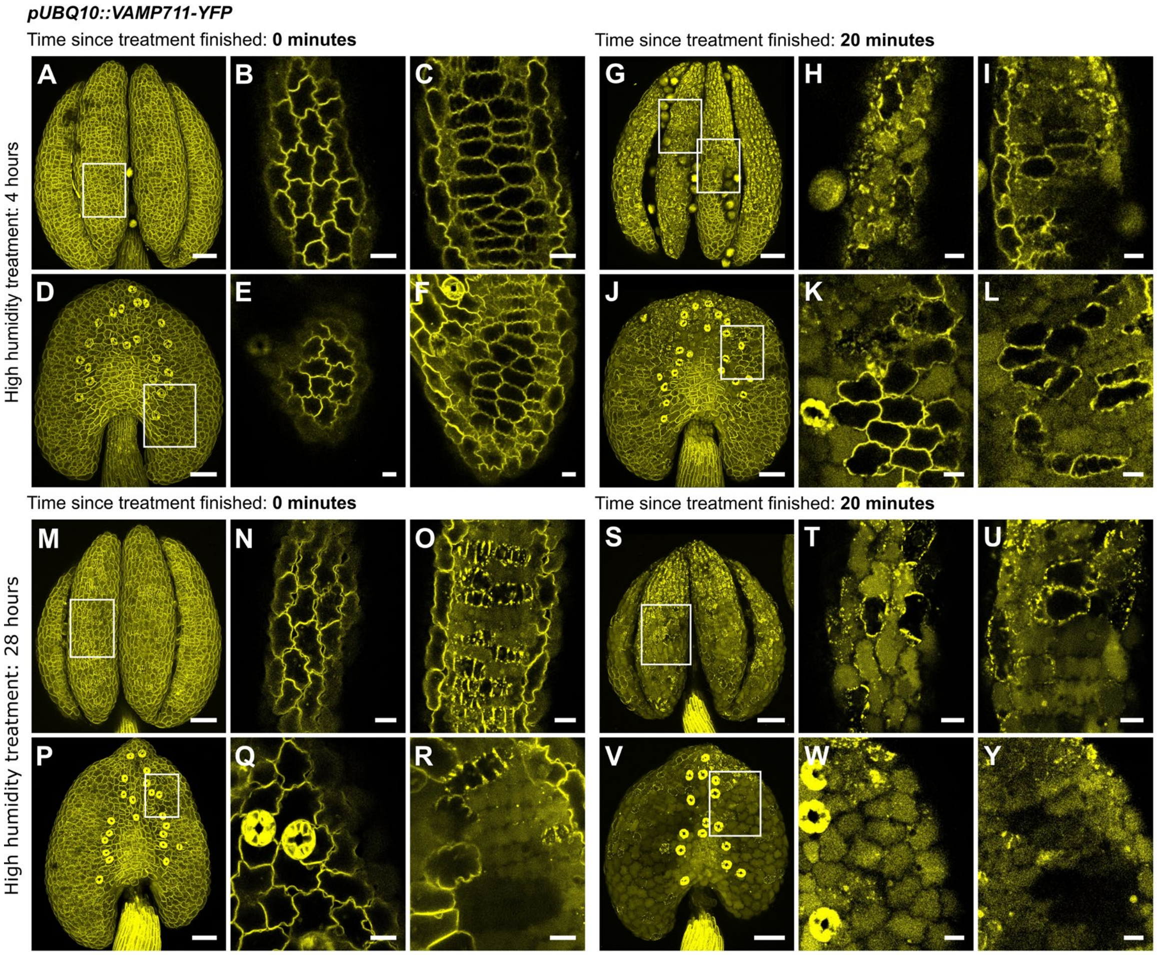

Fig. S8. Tonoplast marker shows endodomain shedding as anther opens. *A. thaliana pUBQ10::VAMP711-YFP* anthers are closed until the opening starts when short and long HH treatment is finished. Tonoplast degradation is visible 20 minutes into anther opening (tonoplast shown in yellow). Open anthers can regain their shape when placed in 2,5% low melting agarose. **A-F.** Closed anthers after a short treatment; tonoplast in both epidermis and endothecium is intact. **G-L.** Open anthers after a short treatment; tonoplast in both epidermis and endothecium is already degraded, but tonoplast of some cells remains intact. **M-R.** Closed anthers after long treatment; tonoplast is intact in the epidermis but not in the endothecium. **S-Y.** Open anthers after a long treatment; tonoplast in both epidermis and endothecium is severely affected. **Legend:** Flowers were exposed to HH for 4 or 28 hours and imaged 0 or 20 minutes after transfer to AH. For each group of figures: the upper row represents the adaxial side, and the lower row represents the abaxial side. From left to right: overall figures, epidermis details, endothecium details. The figures were obtained using Leica TCS SP8. Scale bars are 50 µm in overall view figures, 10 µm in close-up figures.

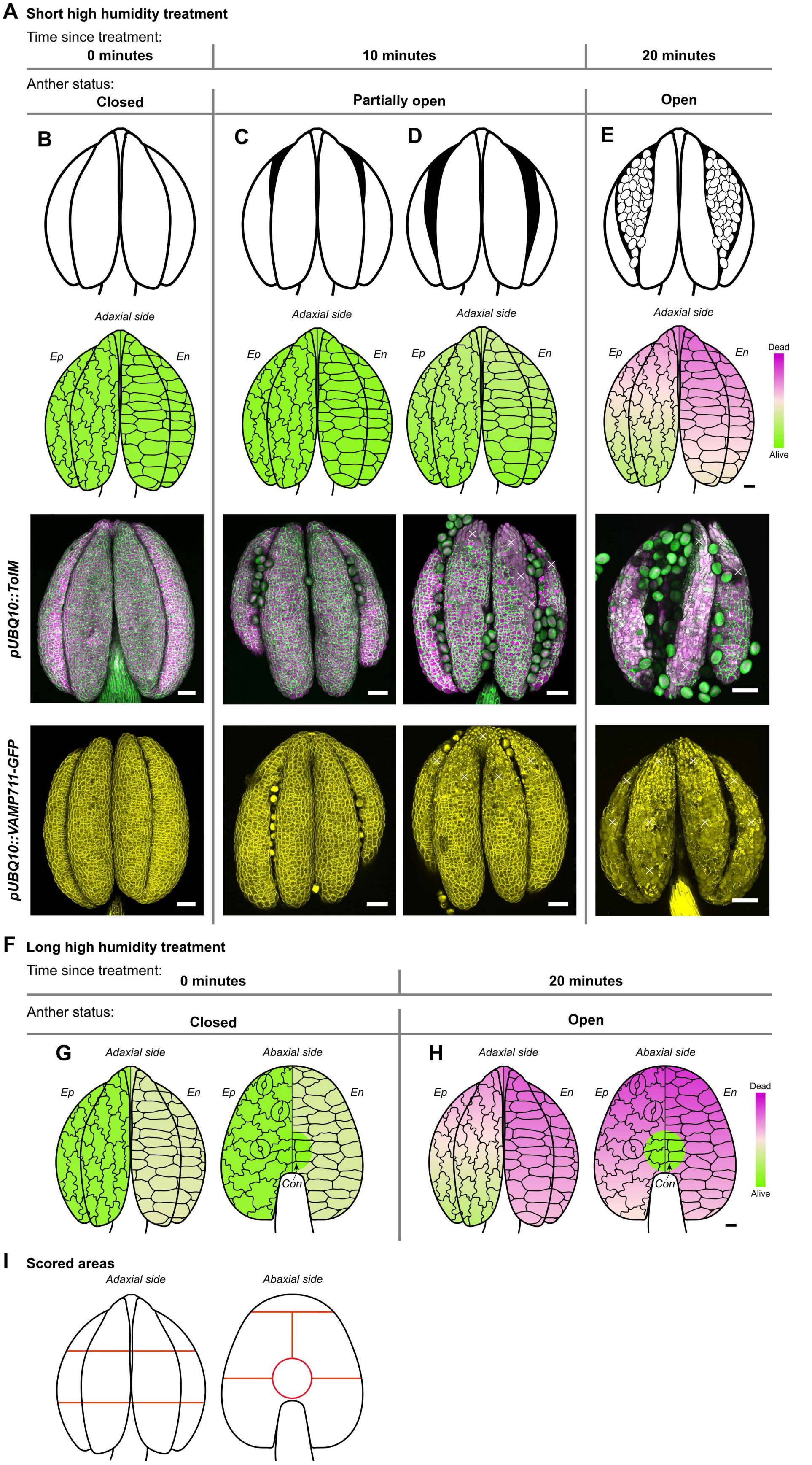

A Short high humidity treatment
Time since treatment:
0 minutes
10 minutes
20 minutes
Anther status:
Closed
Partially open
Open
B
C
D
E
Adaxial side
Ep
En
Dead
Alive
pUBQ10::ToIM
pUBQ10::VAMP711-GFP
F Long high humidity treatment
Time since treatment:
0 minutes
20 minutes
Anther status:
Closed
Open
G
H
Adaxial side
Abaxial side
Con
I Scored areas

Fig. S9. Cell death progresses over time from the anther apex to the base. A total of 144 anthers were analysed from the following *A. thaliana* fluorescent marker lines: *pHTR5::NLS-GFP-GUS, p35S::PIP2-GFP, pUBQ10::ToIM, pUBQ10::VAMP711-YFP, pCEP1::H2A-GFP, pDMP4::H2A-GFP,* and *pSCPL48::H2A-GFP.* Anthers were exposed to (**A**) short (4 hours) or (**F**) long (28 hours) HH treatment and imaged at 0, 10, or 20 minutes after transfer from HH to AH. **A-E. PCD in the adaxial sides of anthers.** The figure is divided into columns, where each contains: 1) a scheme of anther opening status; 2) a color-coded scheme of PCD progression in the epidermis and endothecium (see **Legend**); 3) *pUBQ10::ToIM* anther, where the cytoplasm appears green, the vacuole magenta, and the merged signals indicating cell death result in a lighter magenta colour; and 4) *pUBQ10::VAMP711-YFP* anther, where yellow represents the tonoplast. In 3) and 4), white crosses indicate dead tissue as judged by merged ToIM signals. **B.** Anthers immediately after HH treatment remain closed, and their tissue is intact. **C.** 10 minutes after transfer to AH, anthers begin to open by forming a small slit at the apex, which occurs without detectable PCD in the epidermis and endothecium. **D.** As the slit enlarges, PCD occurs in the epidermis and endothecium. **E.** After 20 minutes in AH following a short HH treatment, anthers are fully open and display advanced PCD. Epidermal cells remain intact longer than endothecial cells, and the anther apex contains more dead cells than the base. **F-H. PCD progression in long HH-treated anthers. G.** Long-treated anthers, adaxial (AD) and abaxial (AB) sides, at time 0. The epidermis is intact, but PCD occurs in the endothecium except for the connective tissue. **H.** Long-treated anthers, AD and AB sides, 20 minutes after treatment. PCD follows a similar pattern to that in short-treated anthers but progresses faster. Connective cells remain viable. **I. Scored areas.** Anthers were divided into several regions indicated by the red lines: apex, middle, and base. The connective area was distinguished on the abaxial side. These areas were scored based on dead cell content: >10 %, 10-40 %, 40-60 %, 60-90 %, and >90 %. **Legend:** Data were analysed using beta regression in R (**betareg** package). Magenta represents completely dead cells, lawn green represents fully living cells, and misty rose represents the transition between dead and living tissue. Each anther scheme has the epidermis (*Ep*) on the left and the endothecium (*En*) on the right. The cell schemes correspond to the assigned tissue of respective anthers on the AD and AB sides. "*Con.*" indicates connective tissue. Cell sizes in the schematics are approximately four times larger than in the anther, scale bar: 10 µm. Figures were acquired using the Leica TCS SP8. The scale bars in the confocal images are 50 µm.

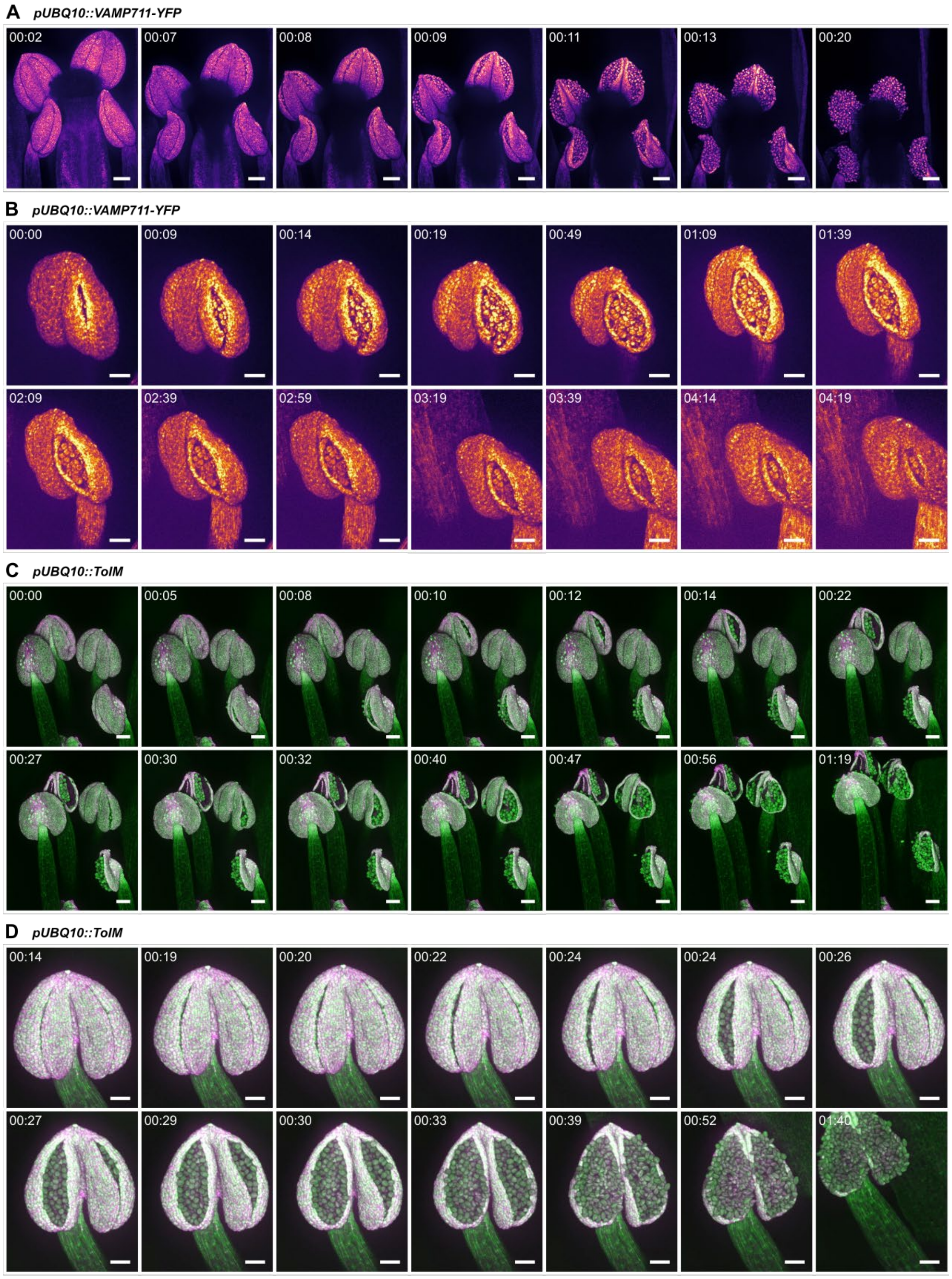

Fig. S10. Anther dehiscence progression can be captured using a spinning disc microscope with a dry mounting approach. This sample preparation enables a completely intact observation

as the stamens remain attached to the plants the whole time. **A-B.** *pUBQ10::VAMP711-YFP* line where tonoplast is shown in magenta-fire. **A.** Anther opening 20 minutes time lapse during which all anthers fully open. **B.** Anther dehiscence time-lapse, 4 hours and 19 minutes, reveals an opening initiation followed by anther closing and regaining the original shape. **C-D.** *pUBQ10::ToIM* line where vacuole is shown in magenta and cytoplasm in green. Signal merging, after the vacuole rupture, is visualised in light pink to white. **C.** The opening time-lapse, 1 hour and 19 minutes, shows the successive opening of all anthers. The filaments continue to elongate even after dehiscence completion. **D.** Time-lapse, 1 hour and 40 minutes, shows anther opening with subsequent filament elongation. All time-lapse series were obtained with a vertical stage(2) spinning disc microscope Zeiss Axio Observer.7/Yokogawa CSU-W1-T2 with a VS-HOM1000 excitation light homogenizer. The resolution was limited due to the dry mounting. Scale bars are 100 µm in A and C and 50 µm in B and D.

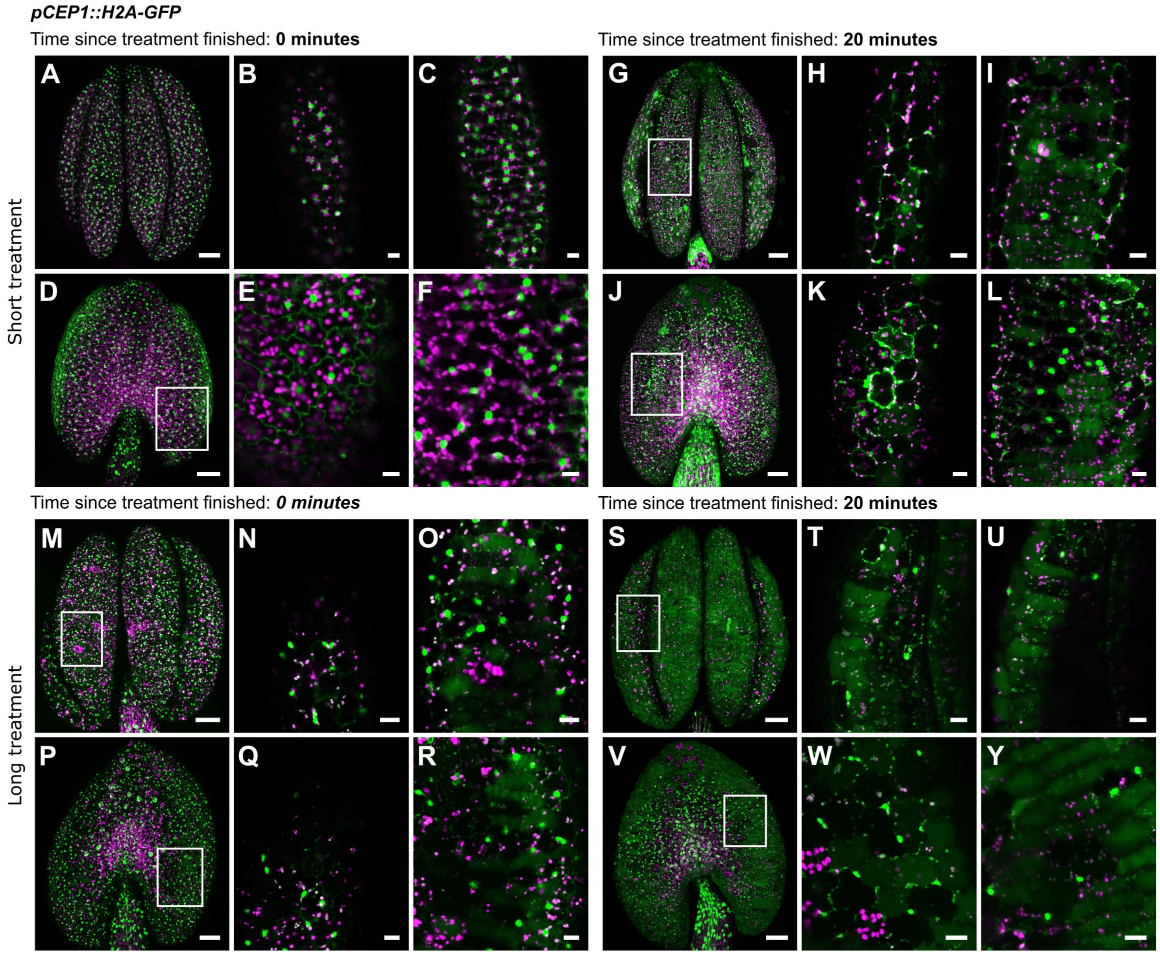

Fig. S11. *A. thaliana pCEP1::H2A-GFP* anthers show cells preparing for PCD, with expression visible in epidermis and endothecium. Nuclei are in green, autofluorescence in magenta. Anthers remain closed during short and long HH treatments, even when the endothecium undergoes PCD during the long treatment. Following treatment completion, anthers open, and cells undergo PCD as the signal spread to the whole cells volume. Already open anthers can partly regain their shape when embedded in 2,5% low melting agarose. **A-F.** Closed anthers after a short treatment; both epidermis and endothecium are fully viable. **G-L.** Open anthers after a short treatment; cells begin undergoing PCD, with some remaining intact. **M-R.** Closed anthers after a long treatment; epidermal cells are mostly intact, while PCD in the endothecium has already commenced. **S-Y.** Open anthers after a long treatment; both epidermis and endothecium exhibit signs of PCD. **Legend:** Flowers were exposed to HH for 4 or 28 hours and imaged 0 or 20 minutes after transfer to AH. For each group of figures, the upper row represents the adaxial side, and the lower row represents the abaxial side. From left to right: overall figures, epidermis details,

endothecium details. The figures were obtained using Leica TCS SP8. Scale bars are 50 μm in overall view figures, 10 μm in close-up figures.

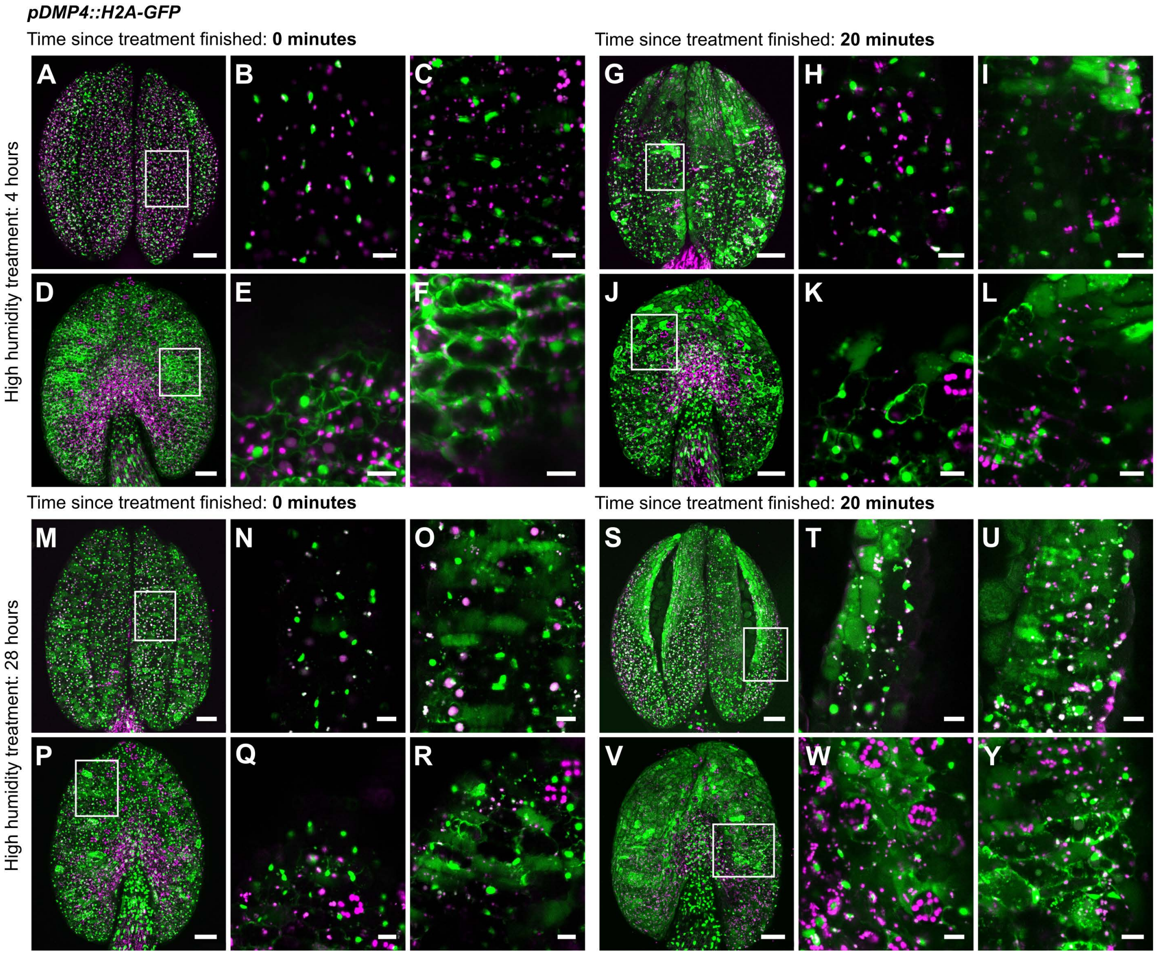

Fig. S12. *A. thaliana pDMP4::H2A-GFP* anthers cells prepare for PCD before opening and then undergo PCD as the anthers open. HH treatments prevent anthers from opening. GFP is shown in green and is restricted primarily to the nuclei in intact cells. Autofluorescence is shown in magenta. As anthers open, the PCD progresses and the nuclei collapse, the signal diffuses and fills the cells. Once open, anthers can regain their shape when placed in 2,5% low melting agarose, but the changes on the cell level are permanent. **A-F.** Closed short-treated anthers; cells are still intact. **G-L.** Open anthers after a short treatment; PCD progresses in both epidermis and endothecium, the apex possesses more dead cells than the base. **M-R.** Closed long-treated anthers; cells are intact in the epidermis; the endothecium contains already dead cells. **S-Y.** Open anthers after a long treatment; most of the cells are dead except for the connective on the abaxial side. PCD again proceeds from the anther apex to the base. **Legend:** Flowers were exposed to HH for 4 or 28 hours and imaged 0 or 20 minutes after transfer to AH. For each group of figures: the upper row represents the adaxial side, and the lower row represents the abaxial side. From left

to right: overall figures, epidermis details, endothecium details. The figures were obtained using Leica TCS SP8. Scale bars are 50 µm in overall view figures, 10 µm in close-up figures.

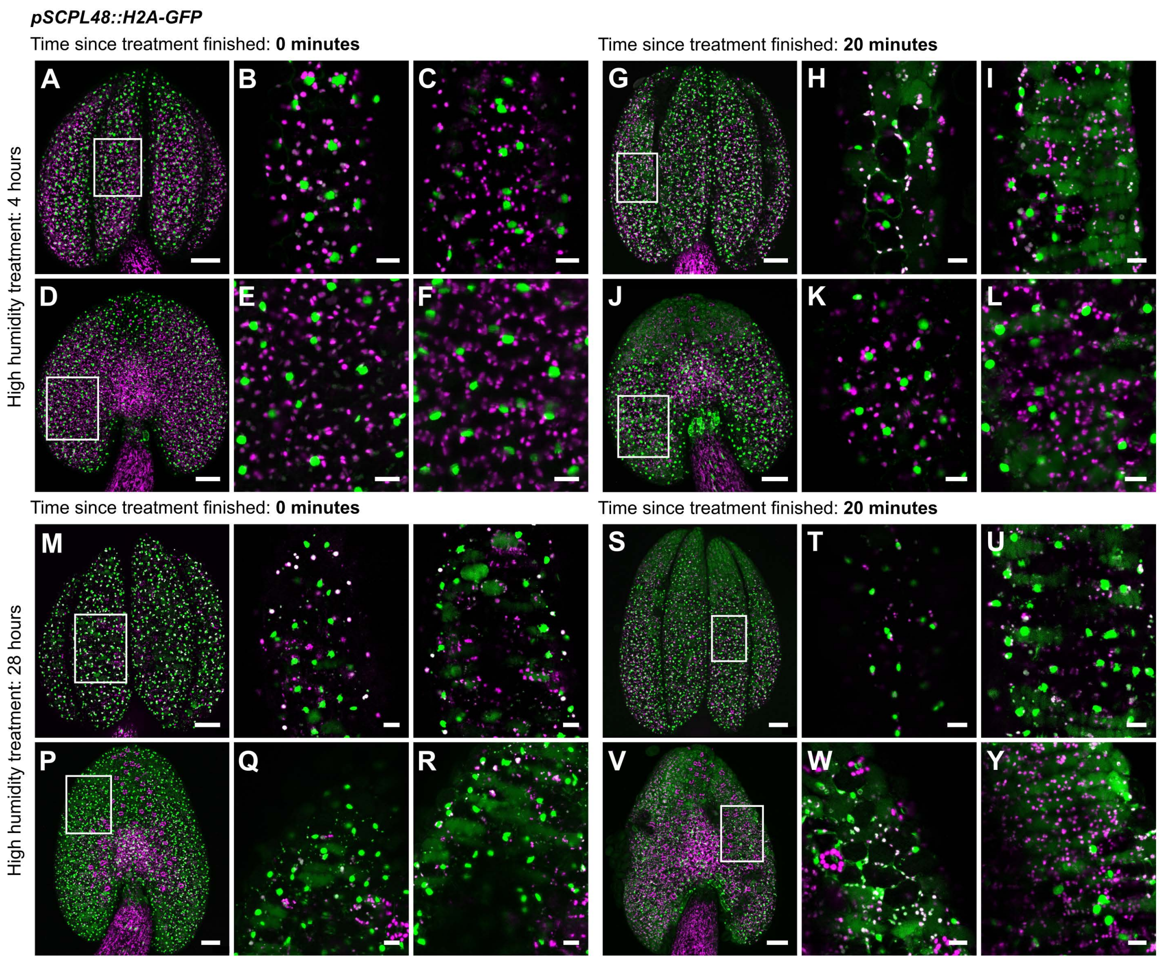

Fig. S13. Anthers of *A. thaliana pSCPL48::H2A-GFP* are pre-programmed for cell death PCD, which is facilitated only once HH treatments end and anthers open. GFP, shown in green, is localised to the nuclei of living cells of closed anthers. During the anther opening, nuclei collapse, cells die, and the signal fills the cells. Autofluorescence is in magenta. Already open anthers can regain their original shape when placed in 2,5% low melting agarose, but the changes on the cell level are definite. **A-F.** Closed short-treated anthers; cells are viable. **G-L.** Open anthers after a short treatment; PCD progresses from the apex to the base, and both epidermis and endothecium are affected. **M-R.** Closed long-treated anthers; epidermal cells are intact, but some endothecial cells are already dead. **S-Y.** Open anthers after a long treatment; cells die in the direction from the apex to the base. Connective tissue stays viable. **Legend:** Flowers were exposed to HH for 4 or 28 hours and imaged 0 or 20 minutes after transfer to AH. For each group of figures: the upper row represents the adaxial side, and the lower row represents the abaxial side. From left to right: overall figures, epidermis details, endothecium details. The figures were obtained using Leica TCS SP8. Scale bars are 50 µm in overall view figures, 10 µm in close-up figures.

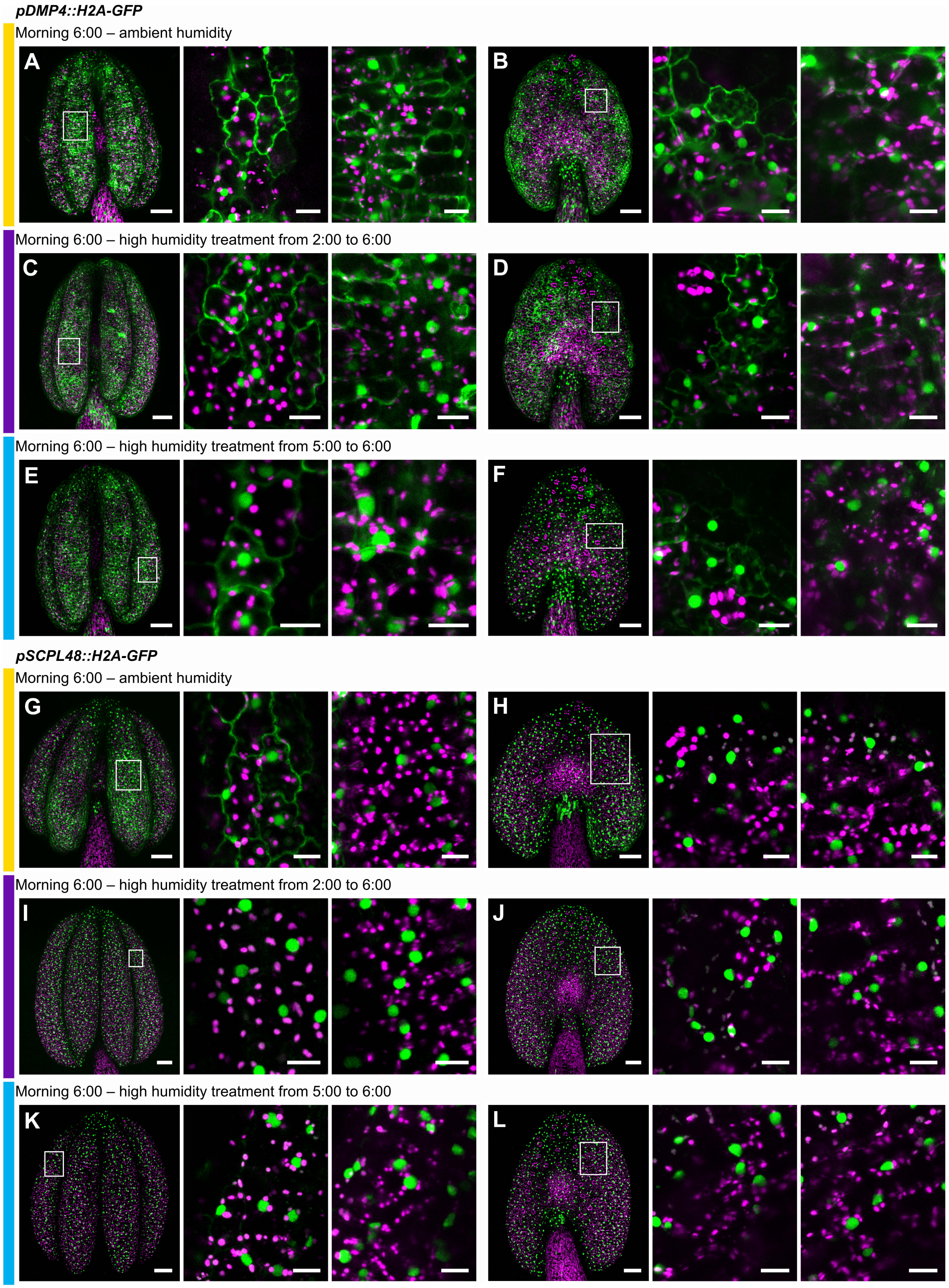

Fig. S14. Analysis of *pDMP4::H2A-GFP* and *pSCPL48::H2A-GFP* in anthers prior to initiation of anther dehiscence indicates dPCD preparation. At AH (yellow), the expression is detected in

still closed anthers at 6:00 in the morning before anther dehiscence, and remains unaffected by preceding exposure HH, whether early HH treatment (starting at 2:00, purple) or standard HH treatment (starting at 5:00, cyan). GFP, shown in green, is seen in the nuclei of living cells of closed anthers. Strong expression results in GFP signal in cytoplasm. Chlorophyll autofluorescence is shown in magenta. **A-F. In *pDMP4::H2A-GFP* anthers, the expression pattern is similar for all three treatments. A-B.** Anthers exposed to AH. **C-D.** Anthers exposed to early HH treatment. **E-F.** Anthers exposed to standard HH treatment. **G-L. In *pSCPL48::H2A-GFP* anthers, all three treatments show a similar expression pattern. G-H.** Anthers exposed to AH. **I-J.** Anthers exposed to early HH treatment. **K-L.** Anthers exposed to standard HH treatment. **Legend:** Plants were placed in AH or flowers were exposed to HH for 1 hour (5:00-6:00) or 4 hours (2:00-6:00). All anthers were examined at 6:00 o'clock in the morning. For each series of figures from left to right: the adaxial side, details of epidermis and endothecium, the abaxial side, details of epidermis and endothecium. Figures were obtained using Leica TCS SP8. Scale bars are 50 μm in the full view figures and 10 μm in the close-up figures.

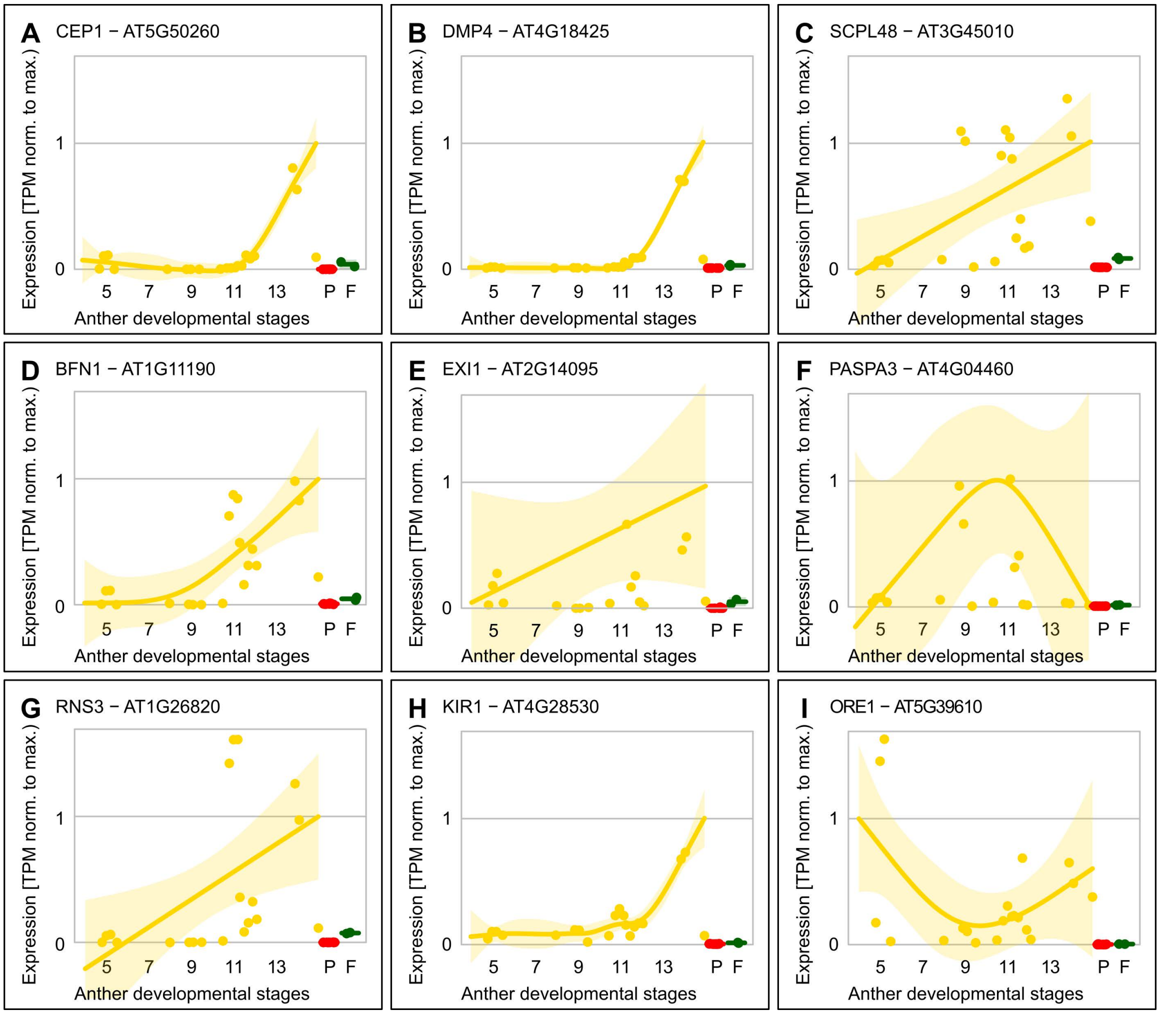

Fig. S15. The expression of studied PCD-related genes *BFN1*, *CEP1*, *DMP4*, *EXI1*, *KIR1*, *ORE1*, *RNS3* and *SCPL48* increases whereas PASPA3 decreases in later anther development. Publicly available RNA-seq data of 12 different developmental phases of anther, 3 independent transcriptomics of mature pollen and one transcriptomic of filament (Dataset S4-5), were quantified using Kallisto 0.48.0 against Araport11 representative CDS model and TPM (transcripts per million) were fitted by GAM (for anther stages) or used for median computation (pollen, filament). In each graph, individual gene transcription (as relative value to fitted maximum) with 95% confidence intervals is shown. Numbers on the x-axis stand for anther development stage according to Sanders *et al.*, 1999(1), P for mature pollen and F for filaments.

Long and short HH treatment of WT *A. thaliana* flowers

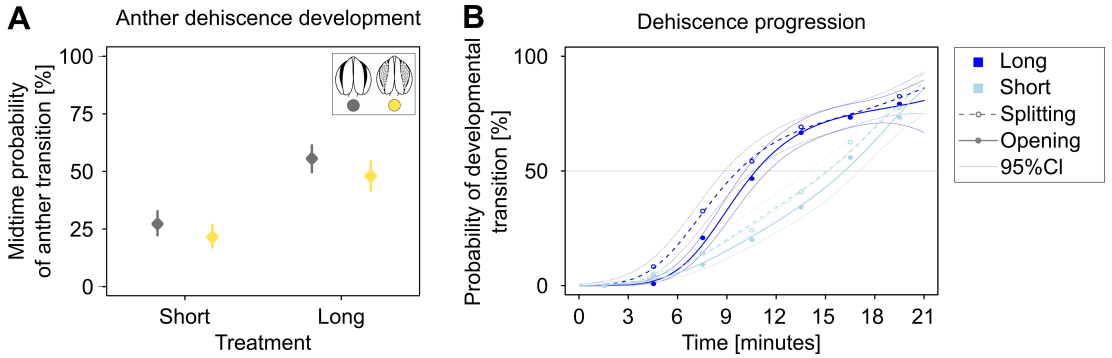

Fig. S16. Anther dehiscence is faster in *A. thaliana* anthers exposed to 28 hours of HH (long HH treatment) compared to short-treated anthers (4 hours). A. Long treatment significantly increases both anther splitting (dark grey) and opening (yellow; GLM, $P < 2.2e\text{-}16$***, 95% CI are displayed). Rates from half-time of the measured period (10.5 minutes) are shown. **B.** The curves show splitting and opening occurring earlier in long treated anthers. Splitting (dashed line) and full opening (solid line) in anthers exposed to long HH treatment (dark blue) compared to the short treatment (light blue). 95% CI are shown.

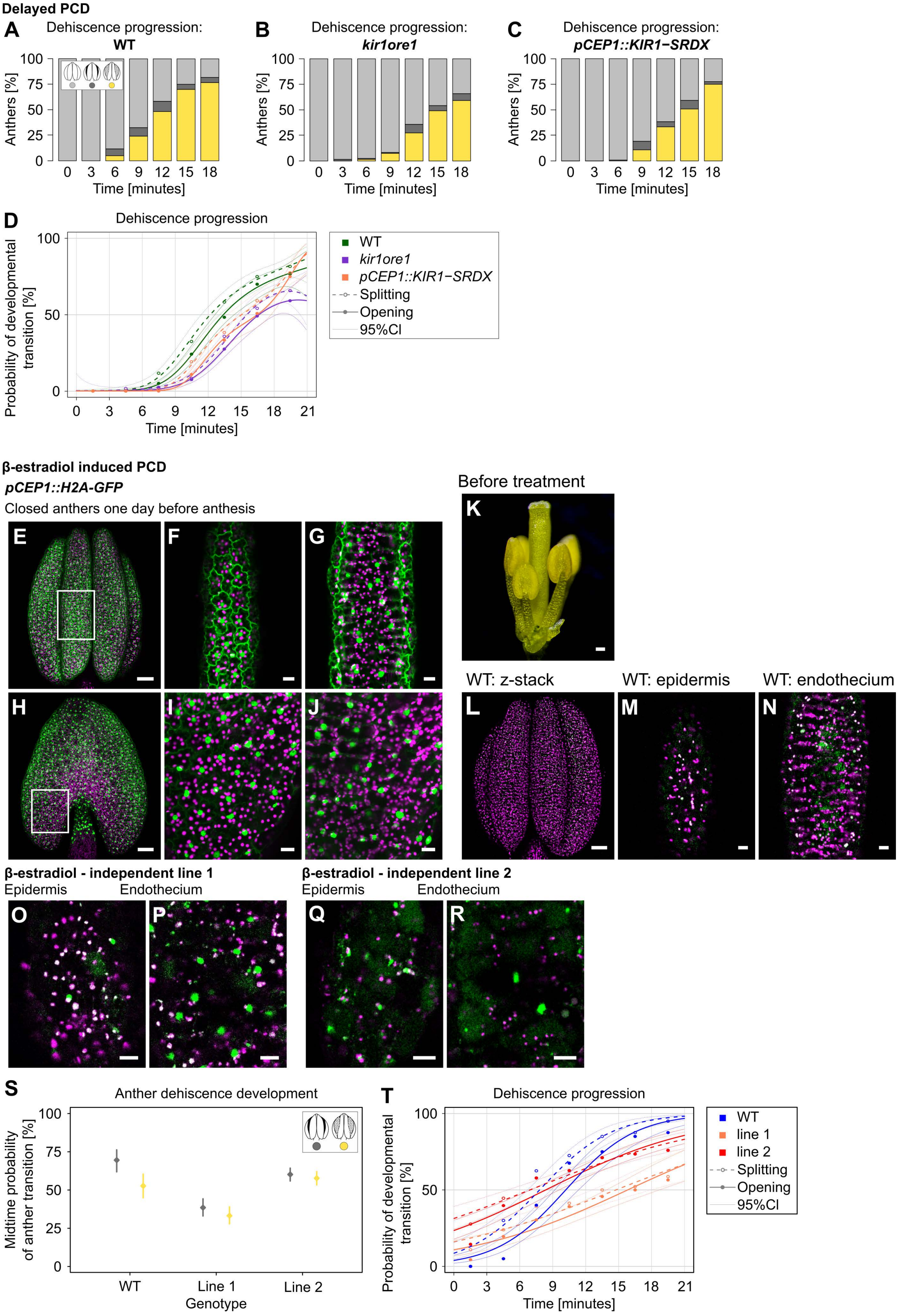
Delayed PCD
A Dehiscence progression: WT
B Dehiscence progression: kir1ore1
C Dehiscence progression: pCEP1::KIR1−SRDX
Anthers [%]
Time [minutes]
D Dehiscence progression
Probability of developmental transition [%]
WT
kir1ore1
pCEP1::KIR1−SRDX
Splitting
Opening
95%CI
β-estradiol induced PCD
pCEP1::H2A-GFP
Closed anthers one day before anthesis
Before treatment
WT: z-stack
WT: epidermis
WT: endothecium
β-estradiol - independent line 1
Epidermis
Endothecium
β-estradiol - independent line 2
Epidermis
Endothecium
S Anther dehiscence development
Midtime probability of anther transition [%]
WT
Line 1
Line 2
Genotype
T Dehiscence progression
Probability of developmental transition [%]
Time [minutes]
WT
line 1
line 2
Splitting
Opening
95%CI

Fig. S17. Genetic manipulation of PCD changes the anther splitting and opening timing. A-D. *pCEP1::KIR1-SRDX* and *kir1ore1* anthers splitting and opening is decelerated when compared to wild-type (WT). Anther dehiscence was measured over 21 minutes in 3-minute steps, all graphs show the dehiscence progression. **A.** WT anther dehiscence, in the midtime, 39 out of 120 are split and open. **B.** *kir1ore1* dehiscence progression, 10 out of 120 are split and open in the midtime. **C.** *pCEP1::KIR1-SRDX* anther dehiscence progression, 23 out of 120 split and open in the midtime. **D.** Probability of anther splitting and opening shows reduced anther dehiscence progression in *pCEP1::KIR1-SRDX* and *kir1ore1* compared to WT (GLM, $P < 2.2e\text{-}16$***, rates and their 95% CI are shown). Legend in the graph. **E-T. Opening despite HH treatment occurs in *pCEP1::XVE≫KIR1-GFP* anthers after PCD is stimulated. E-J.** Expression in the whole tissue occurs in the *pCEP1::H2A-GFP* anthers 24 hours before dehiscence finalisation. This serves as a control that CEP1 is expressed at the time of β-estradiol treatment. **E.** Adaxial side z-stack. **F.** Epidermis on the adaxial side. **G.** Endothecium on adaxial side. **H.** Abaxial side z-stack. **I.** Epidermis on the abaxial side. **J.** Endothecium on the abaxial side. **K.** *pCEP1::XVE≫KIR1-GFP* flower at the beginning of β-estradiol treatment, 24 hours before anthesis. To ensure the β-estradiol solution accessibility, the pistil, sepals and petals are removed (the pistil is kept only for the picture to compare the unmatured stamens length to the pistil length). **L-M.** WT with no induced expression after β-estradiol treatment. **L.** Adaxial side of the anther, z-stack. **M.** Detail of epidermis. Round bodies with overlapping emission spectra (green and magenta resulting in light pink to white) are oil bodies. They are also visible in bright-field z-stack as they are dense and move rapidly in contrast to nuclei. **N.** Detail of endothecium. **O-P.** *pCEP1::XVE≫KIR1-GFP* (independent line 1) 24 hours after β-estradiol induction. **O.** Expression in epidermal cells. **P.** Expression in endothecial cells. **Q-R.** *pCEP1::XVE≫KIR1-GFP* (independent line 2) 24 hours after β-estradiol induction. Tissue already undergoes PCD. **Q.** Expression in epidermal cells. **R.** Expression in endothecial cells. **S-T.** Dehiscence progression was measured in inducible lines and WT as a control over 21 minutes in 3-minute steps (GLM, $P < 2.2e\text{-}16$***, rates and their 95% CI are shown). **S.** Midtime anther splitting and full opening probability differed among all the lines. Legend in the figure. **T.** Probability of anther splitting and full opening shows that anthers of inducible lines open despite the HH. However, the splitting and opening of WT anthers is more rapid over time course. **Legend:** All the fluorescent figures were obtained using Leica TCS SP8. The scale bar in z-stack figures (E, H, L) is 50 µm. The scale bar in close up-figures (F-G, I-J, M-N, O-R) is 10 µm. Figure K was captured with a Nikon D3200 camera and stereomicroscope STM 822 5410, with a scale bar of 100 µm.

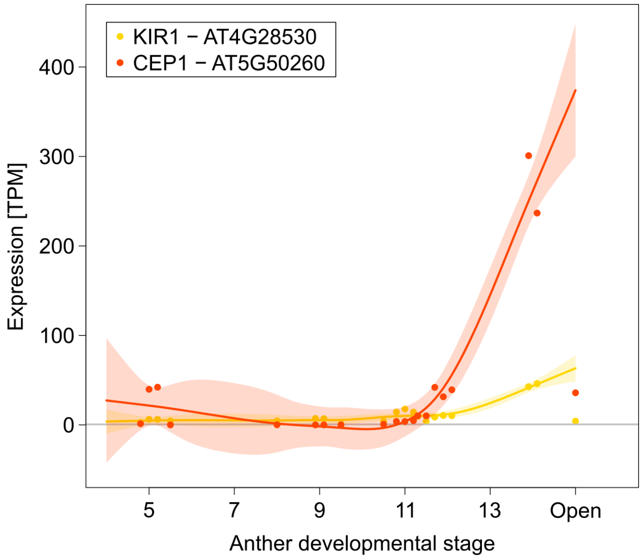

Fig. S18. The expression of PCD-related genes, *CEP1* and *KIR1* from the inducible overexpression line *pCEP1::XVE≫KIR1-GFP*(3), increases prior to anther dehiscence. Publicly available RNA-seq data of different developmental phases of anther (Dataset S4-5) were quantified using Kallisto 0.48.0 against Araport11 representative CDS model and TPM (transcripts per million, y-axis) were fitted by GAM. In the graph, individual genes transcription (expression curves were not normalised to the min-max range) with 95% CI are shown. Individual libraries were not normalized before quantification, quantile-normalized expression profiles gave almost identical expression pattern. Numbers on the x-axis stand for anther development stage according to Sanders *et al.*, 1999(1)**.**

Legend for movie:

Movie S1 (separate file). Using a spinning disc microscope with a dry mounting approach, the progression of anther dehiscence can be recorded. As the stamens remain attached to the plant throughout the process, this sample preparation allows for a fully intact observation. 20-minute time lapse of *A. thaliana pUBQ10::VAMP711-YFP* anther dehiscence is shown, the tonoplast is in magenta-fire. All anthers are fully open at the end of this recording. Time is written mm:ss. The scale bar is 100 µm.

Legends for datasets:

Datasets S1–S12 (separate files).

Name	Description
Dataset S1	Anther opening inhibition by high humidity treatment
Dataset S2	Anther opening in stomata mutants, stomata number and abaxial size analysis
Dataset S3	Progression of anther dehiscence at ambient humidity after abscisic acid (ABA) treatment
Dataset S4	Transcriptomes and RNA-Seq libraries used in this study
Dataset S5	Kallisto expression profiles of all *A. thaliana* representative transcripts in anthers and pollen samples based on public datasets
Dataset S6	Quantification of cell death during anther dehiscence
Dataset S7	Quantification of cell death at first 10 minutes of dehiscence
Dataset S8	Anther opening after long high humidity treatment
Dataset S9	Anther opening in PCD lines
Dataset S10	Key resource table
Dataset S11	PP3 conversion file for green channel
Dataset S12	PP3 conversion file for red channel

SI References

1. P. M. Sanders, *et al.*, Anther developmental defects in Arabidopsis thaliana male-sterile mutants. *Sex. Plant Reprod.* **11**, 297–322 (1999).
2. D. von Wangenheim, *et al.*, Live tracking of moving samples in confocal microscopy for vertically grown roots. *Elife* **6** (2017).
3. Z. Gao, *et al.*, KIRA1 and ORESARA1 terminate flower receptivity by promoting cell death in the stigma of Arabidopsis. *Nat. plants* **4**, 365–375 (2018).
